# Treatment with insulin (analogues) and breast cancer risk in diabetics; a systematic review and meta-analysis of in vitro, animal and human evidence

**DOI:** 10.1186/s13058-015-0611-2

**Published:** 2015-08-05

**Authors:** Heleen K Bronsveld, Bas ter Braak, Øystein Karlstad, Peter Vestergaard, Jakob Starup-Linde, Marloes T Bazelier, Marie L De Bruin, Anthonius de Boer, Christine L E Siezen, Bob van de Water, Jan Willem van der Laan, Marjanka K Schmidt

**Affiliations:** Division of Molecular Pathology, The Netherlands Cancer Institute, Plesmanlaan 121, 1066 CX, Amsterdam, The Netherlands; Division of Toxicology, Leiden Academic Centre for Drug Research, Leiden University, Leiden, The Netherlands; Department of Pharmacoepidemiology, Norwegian Institute of Public Health, Oslo, Norway; Departments of Clinical Medicine and Endocrinology, Aalborg University, Aalborg, Denmark; Department of Endocrinology and Internal Medicine (MEA), Aarhus University Hospital THG, Aarhus, Denmark; Division of Pharmacoepidemiology & Clinical Pharmacology, Utrecht Institute for Pharmaceutical Sciences, Utrecht University, Utrecht, Netherlands; Medicines Evaluation Board (MEB), Utrecht, The Netherlands; Centre for Health Protection, National Institute for Public Health and the Environment (RIVM), Bilthoven, The Netherlands

## Abstract

**Introduction:**

Several studies have suggested that anti-diabetic insulin analogue treatment might increase cancer risk. The aim of this study was to review the postulated association between insulin and insulin analogue treatment and breast cancer development, and plausible mechanisms.

**Method:**

A systematic literature search was performed on breast cell-line, animal and human studies using the key words ‘insulin analogue’ and ‘breast neoplasia’ in MEDLINE at PubMed, EMBASE, and ISI Web of Science databases. A quantitative and qualitative review was performed on the epidemiological data; due to a limited number of reported estimates, a meta-analysis was performed for glargine only. A comprehensive overview was composed for in vitro and animal studies. Protein and gene expression was analysed for the cell lines most frequently used in the included in vitro studies.

**Results:**

In total 16 in vitro, 5 animal, 2 in vivo human and 29 epidemiological papers were included. Insulin AspB10 showed mitogenic properties in vitro and in animal studies. Glargine was the only clinically available insulin analogue for which an increased proliferative potential was found in breast cancer cell lines. However, the pooled analysis of 13 epidemiological studies did not show evidence for an association between insulin glargine treatment and an increased breast cancer risk (HR 1.04; 95 % CI 0.91-1.17; p=0.49) versus no glargine in patients with diabetes mellitus. It has to be taken into account that the number of animal studies was limited, and epidemiological studies were underpowered and suffered from methodological limitations.

**Conclusion:**

There is no compelling evidence that any clinically available insulin analogue (Aspart, Determir, Glargine, Glulisine or Lispro), nor human insulin increases breast cancer risk. Overall, the data suggests that insulin treatment is not involved in breast tumour initiation, but might induce breast tumour progression by up regulating mitogenic signalling pathways.

**Electronic supplementary material:**

The online version of this article (doi:10.1186/s13058-015-0611-2) contains supplementary material, which is available to authorized users.

## Introduction

Breast cancer is the most prevalent cancer in women with 1.67 million new cancer cases diagnosed in 2012 worldwide [1]. Diabetes mellitus (DM) has been associated with breast cancer [2]. However, it is unknown if this association is due to the high blood glucose levels of DM, hyperinsulinaemia, shared risks factors such as obesity, or side-effects of diabetic treatment.

Exogenous insulin treatment for diabetics includes animal insulin, human insulin and insulin analogues. Insulin can act as a growth factor, and it is biologically plausible that use of exogenous insulin (analogues), could stimulate neoplastic growth [3]. The initial source of insulin for clinical use in humans was from animal pancreas. Gradually animal insulin has been almost completely replaced by modified or biosynthetic human insulin, such as NPH, Lente or Regular, and insulin analogues. Insulin analogues have been marketed since 1997 and are different from the human insulin molecule in that the amino acid sequence is modified to have an altered pharmacokinetic profile. These modifications afford greater flexibility in the treatment of diabetic patients. However, structural transformation of human insulin might also result in different binding affinity towards the insulin-like growth factor-1 (IGF-1) receptor (IGF1R). This may result in increased mitogenic action of insulin analogues. As each insulin analogue has different alterations in the amino acid sequence, the pharmacologic properties of the analogues are slightly different. Therefore it could be that various insulin analogues have different tumour promoting properties. Glargine is theoretically most likely to have increased mitogenic action compared to human insulin, as the carboxy terminal of the B-chain of glargine has a positive charge, as is the case with IGF-1.

In 2009, the results of four large-scale epidemiological studies were published, raising the concern that insulin analogues, especially insulin glargine, might increase the risk of cancer [4–8]. Two of these studies suggested that insulin glargine may be associated with a higher risk of cancer than treatment with human insulin [5, 8]. Although the results were inconsistent and the authors stressed the limitations of their studies, this led to an urgent call for more research by the European Association for the Study of Diabetes [9].

Previous reviews that focussed on in vitro studies consistently reported that in contrast to other commercially available analogues, glargine has increased binding affinity towards IGF1R. Most studies concluded that glargine may have increased mitogenic potential in particular at supra-physiological concentrations [10, 11]. Extrapolation of these results to humans is difficult due to obvious limitations of in vitro studies, but also due to tissue-specific biological responses. A focus on a specific cancer type could clarify this issue.

The published animal studies on insulin analogues and cancer have not been reviewed so far. In addition, meta-analyses of epidemiological studies have been inconsistent. One meta-analysis reported an increased relative risk (RR) of any cancer among insulin (analogue) users compared to non-insulin-treated diabetics of 1.39 (95 % CI 1.14, 1.70) [12], while another reported no effect (RR 1.04; 95 % CI 0.75, 1.45) [13]. Insulin use was not associated with an increased risk of breast cancer. However, two [13, 14] out of four meta-analyses [13–16] concluded that the risk of breast cancer was increased among glargine users compared to non-glargine-users.

Considering that cancer is a heterogeneous disease with different aetiologies, and breast cancer being the most common female cancer, we focussed this review on the association of exogenous insulin (analogue) exposure and the risk of breast cancer. To study breast cancer risk in an in vitro, animal and human setting, we made a distinction between tumour initiation and progression as most in vivo and in vitro studies can only address tumour progression. Furthermore, from the literature review we deducted what is currently known about signalling pathways involved in insulin-induced tumourigenesis. We included all widely prescribed insulin analogues and insulin AspB10 and included in vitro, animal, in vivo human and epidemiological studies. To our knowledge, this is the first review to provide a complete overview (including in vitro, in vivo and epidemiological evidence) on whether and how insulin analogues could affect breast cancer risk in diabetic patients.

## Methods

This systematic review is registered at PROSPERO [17] with the registration number: CRD42012002477 and was developed according to the preferred reporting items for systematic reviews and meta-analyses (PRISMA) guidelines [18], and with guidance from the Cochrane Collaboration handbook [19].

### Data sources and searches

A search of MEDLINE at PubMed, EMBASE, and ISI Web of Science, was performed using key words ‘insulin (analogue)’ and ‘breast cancer’ (or similar terms) through July 2014. The full search strategy is described in the electronic supplementary material (Additional file 1: ESM 1).

### Study selection

**Fig. 1:**
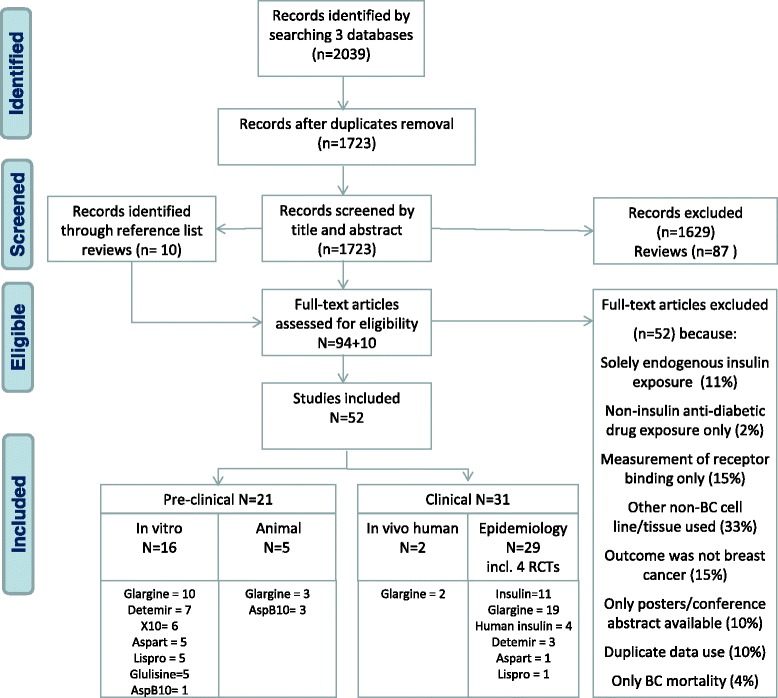
Flow chart of study identification and study selection process. *BC* breast cancer

Eligible studies had to describe effect measures of exogenous insulin (analogue) use on breast cancer development. We included studies with direct (tumour incidence, size, volume, and metastases) or indirect outcomes (cell proliferation, count, and apoptosis, as well as genes and/or proteins explaining mechanisms of breast cancer tumour development e.g., mitogen-activated protein kinase (MAPK), phosphatidylinositol-4,5-bisphosphate 3-kinase (PI3K), phosphatase and tensin homolog (PTEN), mechanistic target of rapamycin mTOR (p53), that are associated with breast cancer. Studies were divided into three categories with the following selection criteria; 1) in vitro studies of mammary gland cell lines exposed to insulin analogues, in which direct proliferative effect was measured or pathway activation was monitored; 2) animal studies on models treated with insulin analogue, in which the mammary gland tumour progression/initiation was measured, or different insulin analogues were compared for their activation of mitogenic signalling pathways in mammary gland tissue, and 3) epidemiological and in vivo studies in humans, including patients with type 1 or type 2 DM treated with insulin analogues before breast cancer diagnosis; cohort and case–control studies as well as randomized controlled trials were included. Only epidemiological studies that presented relative or absolute risk estimates for breast cancer among insulin users were included. Studies that used a non-DM reference population were excluded. In case of multiple publications on the same dataset, we included the study with most complete data. An overview of the study selection is provided in Fig. 1.

### Data extraction

For the in vitro and animal studies information was extracted on the cell (with insulin receptor (INSR):IGF1R status) or animal model (species, tumour subtype), study design (in vitro: assay, starvation method, exposure time, type and refreshment of medium, and presence of phenol red; animal: tissue and proteins analysed, and time of sampling), the intervention (compounds and concentration/dose tested) and the study outcome (mammary tumour formation, mitogenic response, and pathway activation) (Tables 1 and 2).

**Table 1:** Overview of in vitro studies in breast cancer cell lines on the mitogenic potential of insulin analogues

Author, year	Cell line	INSR/IGF1R	Method	Starvation	Stimulation time	Refreshment of medium	Type of stimulation medium	Presence phenol red	Analogues tested	Concentrations tested nM	Mitogenic response	Sig.	PI3K pathway*	MAPK pathway*
Milazzo et al., 1997 [26]	MCF7^A^	1:4	[3H]Thymidine incorporation	Yes	24 hrs stim 2 hrs measure	Yes	MEM DME/F12 + 0.1 % BSA	Yes	AspB10	10	↑^A,B^	Yes		
		1:0.8	DNA measurement	Yes	3−5 days	Yes, every two days	MEM DME/F12 + 0.1 % BSA	Yes	AspB10	0.01−10	↑^A,B^	yes		
	MCF10^B^		Colony forming assay	No	2 weeks	Yes, every two days	MEM DME/F12 + 2 % BSA	Yes	AspB10	100	↑^A^ -^B^	Yes		
Staiger et al., 2007 [32]	MCF7^A^	-	[3H]Thymidine incorporation	48h^A^	20 hrs stim 4 hrs measure	Yes	DME/F12 SFM	No	Glargine	10, 50, 100	↓^A^	No		
	MCF10^B^	-	MTT	24h^B^	4 days	Yes, every two days	DME/F12 SFM	No	Glargine	1, 5, 10, 25	↑^A,B^	No		
				No										
Liefvendahl et al., 2008 [24]	MCF7 SKBR-3	1:20	[3H]Thymidine incorporation	24 hrs	21 hrs stim 3 hrs measure	No	DMEM SFM	No	Glargine	0.01−100	-			
		1:1.8												
Mayer et al., 2008 [25]	MCF7^A^	1:3	Cristal violet cell staining	No	4 days	No	DMEM + 1 % SD-FBS	No	Aspart	1.5^A,B^				
									Lispro	15^A,B^				
	MCF10A^B^	1:1.2							Glargine	1500^C^	↑^A^	Yes^A^		
									Glulisine					
	T47D^C^	1:2							Detemir					
Shukla et al., 2009 [31]	MCF7^A^	-	Cristal violet cell staining	24 hrs	3 days^A^	Yes, every 24 hrs	DMEM + 2 % CDFBS	No	Aspart	1.5, 15, 150,	↑^A^	No		
									Lispro	1500	-			
					2 days^B^		MEGM^B^		Glargine		↑^A^	yes		
									Detemir		↓^A^	No		
	MCF10A^B^	-	WB	24 hrs	10 min	-	DMEM + 2 % CDFBS	No	Aspart				-	-
									Lispro				-	-
							MEGM^B^		Glargine			Yes	↑^A,B^	↑^A^
									Detemir			Yes	↓^A^	-
Shukla et al., 2009 [30]	MCF7^A^	-	Cristal violet cell staining	24 hrs	3 days^A^	Yes, every 24 hrs	DMEM + 2 % CDFBS	No	Glulisine	1.5, 15, 150, 1500	↓^AB^	No		
	MCF10A^B^	-			2 days^B^		MEGM							
			MMOC/ki67 nuclei count	No	3 days	No	Waymouth medium SFM		Glulisine	750	↓	No		
			WB	24 hrs	10 min	-	DMEM + 2 % CDFBS^A^	No	Glulisine			Yes	↓^AB^	↓^AB^
							MEGM^B^							
Weinstein et al., 2010 [35]	MCF7	-	Cell counting	No	72 hrs	Yes every day	DMEM/SFM		Glargine	100	↑	No		
									Detemir		↑	No		
Oleksiewicz et al., 2011 [27]	MCF7	-	FACS	72 hrs	24−30 hrs	No	DMEM + 0.1 % FCS	No	X10	0.074−2	↑	Yes		
			WB	72 hrs	20−40 min	No	DMEM + 0.1 % FCS	No	X10	0.67, 2		Yes	↑	↑
Teng et al., 2011 [33]	MCF7^A^	-	MTT	24 hours	2 days	Yes, every two days	RPMI + 0.5 % CS-FBS	No	Glargine	20−200	↑^A^	Yes		
												Yes		
			WB	No	0, 30, 60, 120, 240 min	No	RPMI + 0.5 % CS-FBS	No	Glargine	100nM	↑^A^			
			FACS anti-apoptotic	No	48 hrs	No	RPMI + 0.5 % CS-FBS		Glargine		↑^A^ anti-Apoptotic response	Yes		
Glendorf et al., 2012 [21]	HMEC	1:20	[3H]Thymidine incorporation	No	70 hrs stim 2 hrs measure	No	MEGM	?	B10A,	0.0001−1000	↓			
									B10R,		↓			
									X10,		↑			
									B10Q,		↑			
									B10E,		↑			
									B10H,		↓			
									B10I,		↓			
									B10F,		↓			
									B10W,		↓			
									B10V		↓			
Hansen et al., 2012 [22]	HMEC^A^	1:21	[3H]Thymidine incorporation	24 hrs	70 hrs stim 2 hrs measure	No	MEGM	No	Detemir	0.001−1000	↓^A^	Yes		
									Glargine		↑^A^	Yes		
									X10		↑^A^	Yes		
Knudsen et al., 2012 [23]	MCF7^A^	-	[3H]Thymidine incorporation	2 hrs	24 hrs stim 2 hrs measure	No	DMEM + 0.1 % serum	No	S961	0.0001−100	↑^A^			
Pierre-Eugene et al., 2012 [28]	MCF7^A^	-	BRET-PIP_3_	No	45 min	No	DMEM/F12 + 5 % FBS	?	Aspart				-	
	MDA-MB-231^B^	-							Lispro				-	
									Glargine			Yes	↑^A^	
									M1				↓^A^	
									M2				↓^A^	
									Glulisine			Yes	↓^A^	
									Detemir			Yes	↓^B^	
			WB	12	5 or 20 min	No	DMEM/F12	?	Glargine				↑^A^	↑^A^
							SFM		M1				-	-
									M2				-	-
			[^14^C]Thymidine incorporation	4 hrs	19 hrs stim 6 hrs measure	No	DMEM/F12 SFM	?	Glargine	0.01−1000	↑^A^			
									M1		-			
									M2		-			
Gallagher et al., 2013 [20]	MET1		WB	1 hr	10 min	No	DMEM + 0.1 % BSA		X10	10	↑	Yes		
	MVT1													
Ter Braak et al., 2014 [34]	MCF7 IGF1R^A^	1:25	WB		30 min	No	RPMI + 5 % CDFBS	No	Aspart	10, 33, 100			-	-
	MCF7 INSR^B^	1:0.02							Lispro			Yes	↑^A^	-
	MCF7 INSR^C^	1:0.07							Glargine			↑^A^	↑^ABC^	
									M1				-	-
									M2				-	-
									Glulisine			-	-	
									Detemir			Yes	↓^AB C^	↓^ABC^
									X10			Yes	↑^A^	↑^ABC^
			SRB	24 hrs	4 days	Yes	RPMI + 5 % CDFBS	No	Aspart	0.01−100	-			
									Lispro		-			
									Glargine		↑	Yes		
									M1		-			
									M2		-			
									Glulisine		-			
									Detemir		↓	Yes		
									X10		↑	Yes		
Sciacca et al., 2014 [29]	MCF7^A^	1:6	BRDU incorporation	24 hrs	12 hrs, 6 hrs measure	No	MEM SFM	?	Aspart	5 nM	↓^A^ –^B,C,D^			
	MDA-MB-	1:2							Lispro	(only detemir	-^A,C,D^ ↑^B^	Yes^B^		
	157^B^								Glargine	at 19 nM)	-^A,C,D^ ↑^B^	Yes^B^		
	MDA-MB-468^C^	1:0.2							M1		-^A,B,D^ ↓^C^			
	T47D^D^	1:8							M2		↑^A^ -^B,D^ ↓^C^			
									Glulisine		-^A,C,D^ ↑^B^	Yes^B^		
									Detemir		-^A,C,D^ ↑^B^	Yes^B^		
									X10		↑^A,B^ –^c,D^	Yes^B^		
			Collagen invasion assay (Boyden chamber technique)	No	18 hrs	No	MEM SFM	?	Aspart		-^A,D,^ ↑^B,C^			
									Lispro		-^A,D^ ↑^B,C^			
									Glargine		↑^A,B,C^ ↓^D^			
									M1		↑^A,C^ –^B,D^			
									M2		-^A,D^ ↑^B,C^			
									Glulisine		↓^A,D^ ↑^B,C^			
									Detemir		↑^A,B,C,D^			
									X10		↑^A,B,C,D^			

^A/B^Often studies used multiple cell lines. ^A, B, C, D^ Specific cell line for cell-line-specific conclusions. *Some studies used a specific experimental setup that allowed discrimination between the involvement of different pathways. For all these studies the p-ERK and p-AKT served as biomarker for activation of mitogen-activated protein kinase (*MAPK*) or phosphatidylinositol-4,5-bisphosphate 3-kinase (*PI3K*), respectively. *IGF1R* insulin-like growth factor-1 receptor, *BRDU* 5-Bromo-2’-deoxyuridine, *RPMI* Roswell Park Memorial Institute medium, *MTT* Microculture Tetrazolium proliferation Assay, *WB* Western Blot, *BRET-PIP* Bioluminescence Resonance Energy Transfer assay in which the phophatidylinositol-3 phosphate (PIP(3)) production was monitored, *SRB* SulfoRhodamine B proliferation assay, *MEGM* Mammary Epithelial Cell Growth Medium, *MEM* Minimum Essential Medium, *SFM* Serum Free Medium, *CDFBS* Charcoal-Dextran-Treated Fetal Bovine Serum, *Sig* Significant.

**Table 2:** Overview of in vivo studies in animals on the correlation of insulin analogues and breast cancer

Author, year	Model	Number of animals per treatment group	Tissues analysed	Time points sampling	Analogues tested	Dose tested nM	Method	Proteins analysed	Carcinogenic potential	Sig.	Tumour characteristics
Stammberger et al., 2002 [37] (re-evaluation in 2012) [38]	Sprague–Dawley rats and Wistar rats and NMRI mice	5−30	No further tumour characterisation	Follow up of 2 years	Glargine	2, 5, 12.5 IU/Kg	Spontaneous mammary gland tumour formation upon treatment		-		MG adenoma, fibroadenoma, adenocarcinoma
Gallagher et al., 2012 [36]	Orthotopic mammary tumour weight and hyperinsulinaemic MKR mice	3−4	Mammary gland	0−25 days	AspB10	12.5 IU/kg	Tumour volume measurement		↑	Yes	
			Lung metastasis			2x/day	Counting lung metastases		↑	No	
							WB receptor activation	p-INSR	↑	Yes	
								p-IGF1R			
								p-Akt	↑	Yes	
								p-Erk	-		
Tennagels et al., 2013 [39]	Female Sprague–Dawley rats	3−4	Mammary gland	60 min	Glargine	12.5, U/kg	WB kinase activation	p-INSR	-		
					AspB10			p-IGF1R	↑	Yes	
Ter Braak et al., 2015 [40]	p53^R270H/+^WAPCre FVB mice	40	Mammary gland tumors	Chronic exposure till MG tumor development	Glargine	12.5-15 IU/kg	Tumour latency time		↑	No	Majority aggressive EMT no correlation pathology and treatment
					AspB10	150-200 IU/kg			↑	Yes	
							WB protein expression profiling	INSR			
								IGF1R,			
								Erk,			
								p-Erk,	↑	Yes	
								Akt,			
								p-Akt,	↑	Yes	
								EGFR,			
								ER,			
								E-cad,			
								N-cad, Her2			

*IGF1R* insulin-like growth factor-1 receptor, *EGFR* epidermal growth factor receptor, *ERK* extracellular signal-related kinase, *ER* oestrogen receptor, *E-cad* E-cadherin, *N-cad* N-cadherin, *Her2* human epithermal growth factor receptor 2

For each epidemiological study, information was extracted on study design and characteristics, i.e., country, source population, data sources, study period, age group, matching variables for case–control studies, DM type and definition, prevalent/incident insulin users, exposure definition, time of exposure definition, mean duration of exposure, latency period and covariates (Additional file 1: Table S2, S3c), and risk estimates for each exposure comparison (Table 3).

**Table 3:** Relative risk estimations for breast cancer among insulin treatment groups and the evaluation of bias and power of the studies

Author, year	Exposure of interest	Exposure comparison group	Cases/controls*** or cases/person-years**** in exposure group (number)	Cases/controls*** or cases/person-years****in comparison group (number)	Risk Ratio**	95 % CI	Risk of bias	Power
Any insulin-no insulin: hazard ratio								
Carstensen et al., 2012 [43]	Insulin users	No insulin users	248/102,500	2,118/627,100	0.96	0.84, 1.09	Moderate	Adequate
Ferrara et al., 2011 [48]	Insulin users	No insulin users	NR	NR	1.0	0.9, 1.2	Moderate	Adequate
Neumann et al., 2012 [60]	Insulin users	No insulin users	NR/NR*	NR/NR*	**0.86**	**0.81**, **0.91**	High	Adequate
Onitilo et al., 2014 [61]	Insulin users	No insulin users	NR/NR*	NR/NR*	0.84	0.58, 1.23	High	Too low
Any insulin-no insulin: odds ratio								
Bodmer et al., 2010a [41]	Insulin users	No insulin users	43/131	262/1,022	NE	NE	High	Too low
Cleveland et al., 2012 [45]	Insulin users	No insulin users	20/16	50/49	1.15	0.40, 3.40	High	Too low
Any insulin-NIAD: hazard ratio								
Currie et al., 2009a [6]	Insulin users	Metformin only	NR/12,640*	NR/34,847*	1.07	0.79, 1.44	Moderate	Too low
Redaniel et al., 2012a [62]	Insulin and NIAD users	Sulfonylurea only users	33/8,233.8	93/27,308.2	1.23	0.63, 2.38	Low	Too low
Redaniel et al., 2012b [62]	Insulin only users	Sulfonylurea only users	8/2,247.3	93/27,308.2	1.67	0.70, 3.99	Low	Too low
Vallarino et al., 2013****** [67]	Pioglitzone users, not using insulin	Insulin users, not using pioglitazone	181/29,721	113/13,680	0.85	0.67, 1.08	High	Low
Any insulin-NIAD: odds ratio								
Hsieh et al., 2012 [53]	Insulin only users	Metformin only users	5/NR	19/NR	1.63	0.60, 4.40	High	Too low
Koro et al., 2007a [54]	Insulin and NIAD users	TZD users	13/52	83/449	0.71	0.36, 1.37	High	Too low
Koro et al., 2007b [54]	Insulin only users	TZD users	9/62	83/449	1.27	0.61, 2.67	High	Too low
Glargine-no glargine: hazard ratio								
Bordeleau et al., 2014***** [42]	Glargine users	Standard care, not using glargine	28/11,620*	28/12,845*	1.15	0.67, 1.97	Low	Too low
Home and Lagarenne, 2009***** [52]	Glargine users	Any anti-diabetic drug, NPH in 20 studies	4/4,711	6/4,524	0.62	0.17, 2.18	Moderate	Too low
Rosenstock et al., 2009 [63]	Glargine users	NPH users	3/2,144	5/2,096	0.90	0.64, 1.26	Low	Too low
Chang et al., 2011***** [44]	Glargine users, not using int-/long-acting HI	Non-glargine int/long-acting HI users	6/6,558.8*	65/47,724.6*	0.53	0.21, 1.31	Moderate	Too low
Colhoun et al., 2009a [5]	Glargine plus non-glargine insulin users	Non-glargine insulin users	0/NR	29/9,667*	NE	NE	High	Too low
Colhoun et al., 2009b***** [5]	Glargine only users	Non-glargine insulin users	6/1,200*	29/9,667*	1.47	0.59, 3.64	High	Too low
Currie et al., 2009b***** [6]	Glargine users	Non-glargine insulin users	10/2,245*	38/8,102*	0.86	0.42, 1.75	Moderate	Too low
Fagot et al., 2013a***** [47]	Glargine users	Other int-/long-acting insulin only users	114/42,129*	40/14,082*	1.08	0.72, 1.62	High	Too low
Habel et al., 2013a**** * [51]	Glargine users	NPH insulin users	52/10,614.8	217/60,868.1	1.3	1.0, 1.8	Moderate	Too low
Habel et al., 2013b [51]	Glargine only users	NPH insulin users	33/6,402.4	217/60,868.1	1.3	0.9, 2.0	Moderate	Too low
Habel et al., 2013c [51]	Glargine and NPH insulin users	NPH insulin users	19/4,212.5	217/60,868.1	1.3	0.8, 2.0	Moderate	Too low
Kostev et al., 2012a***** [55]	Glargine users	NPH insulin users	NR	NR	0.93	0.68, 1.27	High	Too low
Lind et al., 2012a***** [56]	Glargine users	Non-glargine users	19/7,019.4	96/48,889.6*	1.54	0.90, 2.67	Moderate	Too low
Morden et al., 2011a [59]	Glargine plus non-glargine insulin users	Non-glargine insulin users	102/18,889*	333/65,294*	1.08	0.86, 1.36	High	Low
Morden et al., 2011b***** [59]	Glargine only users	Non-glargine insulin users	118/21,071*	333/65,294*	1.03	0.83, 1.29	High	Low
Ruiter et al., 2012a***** [64]	Glargine only users	Human insulin only users	11/6,875*	NR; IR=2.28*	**1.65**	**1.10, 2.47**	Moderate	Too low
Sturmer et al., 2013a **** [65]	Glargine users	NPH users	103/26,277	19/5,885	1.07	0.65, 1.75	Moderate	Too low
Suissa et al., 2011a***** [66]	Glargine users	Non-glargine insulin users	18/6,094	60/12,262	0.8	0.3, 2.1	Moderate	Too low
**Pooled hazard ratio**	Glargine	No glargine			1.04	0.91, 1.17		
Glargine-no glargine: incidence rate ratio								
Ljung et al., 2011a [57]	Glargine plus non-glargine insulin users	Non-glargine insulin users	59/25,033	283/101,419	1.04	0.77, 1.41	High	Low
Ljung et al., 2011b [57]	Glargine only users	Non-glargine insulin users	31/7,302	283/101,419	**1.58**	**1.09, 2.29**	High	Too low
Glargine-no glargine: odds ratio								
Grimaldi-Bensouda et al., 2013a [49]	Glargine users	Non-glargine users	78/287	697/2,763*	1.04	0.76, 1.44	Low	Borderline
Grimaldi-Bensouda et al., 2013b [49]	Glargine users	Non-glargine insulin users	74/203	70/207	0.96	0.61, 1.53	Low	Too low
Grimaldi-Bensouda et al., 2013c [49]	Glargine users	Human insulin users	NR	NR	1.29	0.78, 2.13	Low	NE
Grimaldi-Bensouda et al., 2013d [49]	Glargine users	Aspart users	NR	NR	1.10	0.64, 1.89	Low	NE
Grimaldi-Bensouda et al., 2013^e^ [49]	Glargine users	Lispro users	NR	NR	0.85	0.48, 1.50	Low	NE
Mannucci et al., 2010a [58]	Glargine users	Non-glargine insulin users	NR	NR	NE	NE	High	Too low
Determir-no determir: hazard ratio								
Fagot et al., 2013b [47]	Determir users	Other int-/long-acting insulin only users	38/12,806*	116/43,131*	1.08	0.72, 1.62	High	Too low
Kostev et al., 2012b [55]	Detemir users	NPH insulin users	NR/789	NR/4,206	1.17	0.66, 2.06	High	Too low
Determir-no determir: incidence rate ratio								
Dejgaard et al., 2009a [46]	Determir users	NPH users	1/2,252	0/1,420	NE	NE	Low	Too low
Dejgaard et al., 2009b [46]	Determir users	Glargine users	1/917	3/628	NR	NR	Low	Too low
Aspart-no aspart: odds ratio								
Grimaldi-Bensouda et al., 2013f [55]	Aspart users	Non-aspart users	54/241	721/2,809*	0.95	0.64, 1.40	Low	Borderline
Lispro-no lispro: odds ratio								
Grimaldi-Bensouda et al., 2013g [49]	Lispro users	Non-lispro users	46/133	729/2,917*	1.23	0.79, 1.92	Low	Borderline
Human insulin-no human insulin: hazard ratio								
Fagot et al., 2013c [47]	Basal human insulin users	Other int-/long-acting insulin only users	15/5,813*	139/50,948*	1.03	0.56, 1.88	High	Too low
Gu et al., 2013 [50]	Human insulin users	No insulin users	4/6,188*	14/10,435*	0.33	0.10, 1.13	Moderate	Too low
Ruiter et al., 2012b [64]	Non-glargine insulin users	Human insulin only users	31/15,578*	NR; IR=2.28*	0.99	0.81, 1.20	Moderate	Too low
Human insulin-no human insulin: odds ratio								
Grimaldi-Bensouda et al., 2013h [49]	Human insulin users	Non-human insulin users	59/260	716/2,790*	0.81	0.55, 1.20	Low	Borderline

Bold values are significantly different. *Calculated using data provided (if not indicated directly these were taken from the table in the paper). **Risk estimates are adjusted for covariates as stated in Additional file 1: Table S3. Covariates used in the various analyses are the same within one study. ***Case–control studies. ****Cohort studies or randomized clinical trials. *****Included in meta-analysis. ******The exposure of interest is the exposure comparison group in this analysis. Studies are first ordered by type of exposure and then by type of risk estimate. Note: Hiesh 2012 is a cohort study but provided odds ratio estimates in the paper. Names of exposure groups are defined by the authors of the study. Several papers showed multiple risk estimates for the same exposure with different analytical approaches. For each study and exposure, the results from the least biased or best performed analyses are shown; showing hazard ratios, incidence rate ratios or odds ratios as applicable. Different exposure comparisons within one study are indicated by a,b,c etc. We choose to include the risk estimate that gave (in order of importance): 1) estimates for incident users was preferred over estimates for prevalent users; 2) as-treated analysis (during study period/follow up) was preferred over intention-to-treat analysis (during fixed period/at baseline); 3) estimates with the longest latency period were preferred. Estimates from statistical models adjusted for covariates were preferred over crude estimate. *NR* not reported, *NE* not estimated, *HI* human insulin, *TZD* Thiazolidinedione, *NIAD* non-insulin anti-diabetic drug, *NPH* Neutral Protamine Hagedorn, *Int* intermediate.

### Data synthesis and analyses

In vitro and animal studies were grouped by type of insulin analogue, and common pathways/mechanisms of action were extracted and summarized. Plausible pathways were suggested based on the strength of the evidence. To substantiate the results of the in vitro studies included in this systematic review, we created an overview of the protein and gene expression in eight commonly used mammary (tumour) cell lines of hormone receptor levels (INSR, IGF1R, ER, PR, HER2, EGFR) and some proteins essential for insulin-induced downstream signalling cascades. The methods of these experiments can be found in Additional file 1: ESM 2.

The exposure comparisons that were examined in the epidemiological studies were categorized as: 1) use of any exogenous insulin versus no use of any exogenous insulin (drug exposure undefined); 2) use of any exogenous insulin versus use of non-insulin anti-diabetic drug (NIAD) (type of NIAD defined); 3) use of insulin X versus no use of insulin X. Results were categorized on the exposure of interest. Data was ordered per risk estimate (hazard ratio (HR), odds ratio (OR), incidence rate ratio (IRR)). If a study presented results within the same exposure comparison, but with different definitions of the exposure of interest (e.g., glargine users or glargine only users), the group that had most power was included to calculate the pooled estimate. We set a subjective cutoff of 10 studies needed for a pooled analysis; hence, this was only performed for glargine. The pooled estimate was derived using the random effect model. Pooled analysis by dose or duration was not feasible, as risk estimates were reported for different exposure comparisons, exposure definitions (e.g., mean or cumulative dose, duration since start exposure, or cumulative duration) and stratification categories. The quality evaluation of the epidemiological studies focussed on potential selection bias, information bias, and confounding. The evaluation process of the bias and power of studies is displayed in Additional file 1: ESM 3. Data were prepared in Microsoft Access 2010 and analysed in Stata version 11.0.

## Results

A search in MEDLINE at PubMed, EMBASE, and ISI Web of Science identified 1,723 unique records (Fig. 1). After the eligibility assessment, 52 studies on exogenous insulin (analogue) exposure and breast cancer were included, of which there were 16 in vitro, 5 animal, 2 human in vivo and 29 epidemiological studies (see Additional file 1: ESM 4 for study descriptions).

### Evidence of mitogenic/carcinogenic potential

**Fig. 2:**
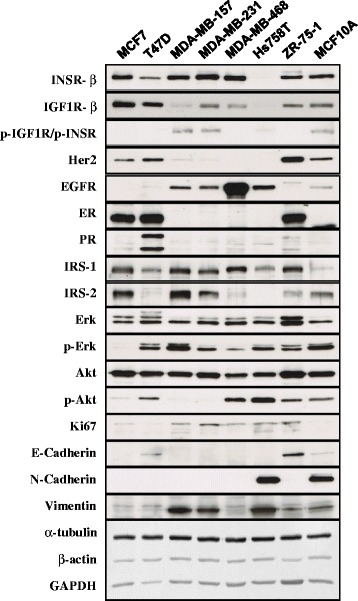
Protein expression profiling of eight commonly used human breast cell lines. Receptor levels and signalling molecules downstream of the insulin receptor/insulin-like growth factor-1 receptor (*INSR*/*IGF1R*) signalling pathway have been quantified. Furthermore some breast cancer subtype markers have been used to further characterize these cell lines that are commonly used in the research articles discussed in this review. *Her2* human epidermal growth factor receptor 2, *EGFR* epidermal growth factor receptor, *ER* oestrogen receptor, *PR* progesterone receptor, *Erk* extracellular signal-related kinase, *GAPDH* glyceraldehyde-3-phosphate dehydrogenase *IRS* Insulin Receptor Substrate

**Fig. 3:**
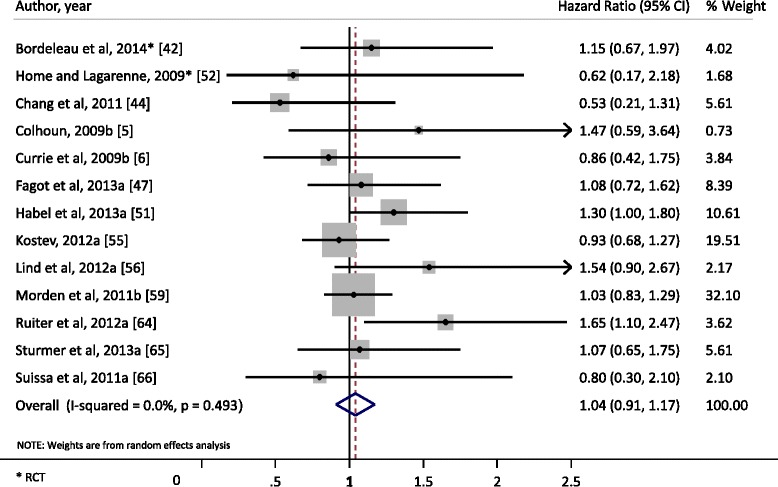
Forest plot of reported hazard ratios for risk of breast cancer among insulin glargine users. *RCT* randomized controlled trial

Current evidence of the mitogenic/carcinogenic potential per insulin (analogue) is described below, highlighting the most important findings displayed in the tables and figures. In Table 1 an overview is presented of all in vitro studies in which the mitogenic potency and/or stimulation of the signalling pathways MAPK and PI3K upon insulin analogue(s) exposure was determined in a mammary gland (tumour) cell line [20–35]. Protein expression of hormone receptors and some downstream signalling proteins for each cell line are provided in Additional file 1: Table S1 and Fig. 2. In Table 2 an overview is presented of all relevant animal studies [36–40]. Descriptions and characteristics of the epidemiological studies are presented in Additional file 1: Table S2, S3c [5, 6, 41–67]. Table 3 lists the overall risk estimates for breast cancer per insulin analogue in the epidemiological studies; the corresponding forest plots are presented in Additional file 1: Figure S1. Results of the meta-analysis on glargine can be found in Fig. 3. Some studies provided risk estimates by strata of duration or dose of exposure (Additional file 1: Table S4). The quality assessment of the epidemiological studies is shown in Additional file 1: Table S5.

#### Insulin glargine (M1/M2)

Seven of ten in vitro studies found an increased proliferative potential of glargine in comparison with human insulin [22, 25, 28, 29, 31, 34, 35] (Table 1). Two studies found proliferation of glargine as well, but human insulin was not included as a reference compound, therefore they could not confirm an increased proliferative response [32, 33]. One study is difficult to interpret, because IGF1 did not show increased mitogenic potential either [24]. Similar to insulin AspB10, glargine has an increased binding affinity towards IGF1R [68]. This receptor is assumed to be responsible for the increased mitogenic action. Studies including kinase activation assays indicate that the PI3K signalling cascade is significantly upregulated after glargine stimulation compared to human insulin stimulation [28, 31, 33, 34]. Two studies also found the MAPK signalling cascade to be upregulated [28, 31]. The clinical relevance of this increased mitogenic potential is as yet unknown because glargine is rapidly metabolised in vivo into two metabolically active compounds, M1 and M2 [69, 70]. These metabolites possess low mitogenic signalling [28, 34].

In a 2-year follow up study, wild-type Sprague–Dawley rats, Wistar rats, and NMRI mice have been used to test the effect of chronic glargine injections compared to the insulin NPH injections; no difference in tumour-free survival was observed [37, 38] (Table 2). In contrast, a recent study revealed a (non-significant) decrease in tumour latency time after a similar chronic exposure to glargine; tumour multiplicity or metastases were not affected [40]. Glargine injections induced no increased receptor activation response in the mammary glands of Sprague–Dawley rats [39].

Three randomized clinical trials (RCT) that investigated breast cancer risk among glargine users compared to non-glargine users [42, 52, 63] did not show significant differences (Table 3). Most case–control and cohort studies showed a non-significant increased risk. Only two observational studies [57, 64] showed a statistically significant increased risk of breast cancer with an IRR of 1.58 (95 % CI 1.09, 2.29) and HR of 1.65 (95 % CI 1.10, 2.47), respectively. Both studies included glargine-only users and compared them to non-glargine insulin users [57] and human-insulin-only users [64]. As the glargine studies did not show statistically significant heterogeneity (*I*^2^ = 0.0 %; p >0.05) a meta-analysis was performed. From 13 studies the pooled HR for glargine vs no use of glargine was 1.04 (95 % CI 0.91, 1.17; *p* = 0.49) (Fig. 3 and Table 3), showing no evidence for an association between insulin glargine treatment and increased incidence of breast cancer.

#### Insulin detemir

Like glargine, detemir is a long-acting insulin analogue. In general, it is assumed that detemir has a lower mitogenic potential compared to human insulin [22, 28, 31, 34], but in a number of in vitro studies similar proliferation or even increased proliferation of determir has been observed [25, 29, 35] (Table 1). The binding characteristics of detemir to albumin are different among species. In almost all in vitro studies BSA or FBS is added to the stimulation medium. Interpretation of these mitogenicity studies is difficult because it is not yet known how the bovine albumin interacts with detemir compared to human albumin [11]. For the same reason it is not surprising that no chronic animal studies have been conducted with insulin detemir. Only three epidemiological studies have been performed, one RCT [46] and two cohort studies [47, 55]; none found an association with breast cancer development (Table 3).

#### Insulin aspart, glulisine and lispro

Compared to glargine and detemir, the insulin analogues aspart, glulisine and lispro are less well evaluated for mitogenic potential; no increased mitogenic action was found in four in vitro studies [25, 28, 30, 34] (Table 1). Only one in vitro study suggested a small non-significant proliferative increase of aspart compared to human insulin [31]. Another in vitro study found the mitogenic potential of glulisine to be significantly lower than human insulin [30]. Evidence that lispro and glulisine had increased proliferative potential was found in just one in vitro study and for just two of the tested cell lines (MDA-MB-157 and MDA-MB-468) [29]. We previously found that the PI3K signalling cascade is significantly more upregulated after lispro treatment than human insulin stimulation only in the IGF1R overexpressing MCF7 cell line [34]. Similar to the in vitro data, the epidemiological data on these short-acting insulin analogues is scarce. Just one study reports ORs for aspart and lispro of 0.95 (95 % CI 0.64, 1.40) and 1.23 (95 % CI 0.79, 1.92), respectively [49] (Table 3).

#### Human insulin

In vitro studies showed that treatment of diabetics with human insulin has low mitogenic potential (Table 1). From the in vivo studies it can be concluded that human insulin is not carcinogenic as the number of tumours that developed in the human-insulin-treated group was similar to the vehicle-injected group (Table 2). Only three epidemiological studies explored the effect of human insulin as the exposure of interest on the risk of breast cancer. Two of these studies compared human insulin users with insulin analogue users [47, 49] and found no significant difference in breast cancer risk (Table 3). The other study compared human insulin users with diabetics not treated with insulin and reported a HR of 0.33 with a relatively wide 95 % CI of 0.10, 1.13 [50]. This study was under powered.

Human insulin, especially NPH, was often used as exposure comparison group in the studies that investigated risk of breast cancer related to insulin analogue use. Most of these studies did not report significant differences in the risk of breast cancer, as mentioned previously.

#### Insulin AspB10

The increased carcinogenic effect of insulin AspB10 had already been discovered in 1992 [71]. Since then this insulin analogue has been used in many in vitro studies as a reference compound with a strong carcinogenic potential. In proliferation studies AspB10 was highly mitogenic compared to human insulin, irrespective of the cell line used [21, 22, 26, 27, 29, 34] (Table 1). Most studies indicated that AspB10 induces proliferation by increased IGF1R signalling, but there are indications that the INSR is also involved because increased proliferation was not fully blocked when using a specific IGF1R inhibitor [26]. One study used two murine mammary tumour cell lines, both expressing INSR and IGF1R. These cell lines were stimulated with AspB10 and only activation of INSR and not IGF1R was observed [20]. In a different study it was indicated that a prolonged occupancy time of this analogue towards the INSR results in sustained activation of this receptor and subsequently increased mitogenic potency [22]. With a collagen invasion assay it was determined in several breast cancer cell lines that AspB10 has an increased invasive capacity compared to human insulin [29]. In a very elaborate kinase/inhibitor study it was found that multiple core kinases are involved in the mitogenic action of AspB10, because phosphorylation of AKT, p70S6K, S6, and 4E-BP1 was found to be increased compared to human insulin exposure [27].

In animal studies*,* AspB10 was found to have dose-dependent increased carcinogenic potential [71] (Table 2). Xenograft rodent models with injected mammary gland tumour cell lines were treated with either human insulin or AspB10. Tumours were significantly bigger after the AspB10 injections and, although not significant, more lung metastases were found in this treatment group. Strong upregulation of p-AKT has been observed on kinase activation analysis of these tumours, indicating that the carcinogenic effects of AspB10 might be a direct effect from a PI3K response [20]. A very recent study used a p53^R270H/+^WAPCre mouse model, which develops spontaneous human relevant mammary gland tumours within 70 weeks, to show that chronic exposure to AspB10 significantly decreases the tumour latency time. A detailed protein expression analysis showed that tumours induced by AspB10 or IGF1 have a distinct expression pattern compared to tumours from insulin- or vehicle-treated mice; both the PI3K and the MAPK were found to be significantly upregulated after AspB10 and IGF1 treatment [40]. A different study focussed on the short term mitogenic effects of AspB10 and found significantly stronger receptor activation in the mammary glands of Sprague–Dawley rats one hour after AspB10 injections compared to human insulin treatment [39]. As insulin AspB10 has been shown to have mitogenic properties in in vitro and animal studies, this drug has never been available to humans.

#### Insulin (analogue) users versus non-insulin users or NIAD users

In the epidemiological studies, the risk of breast cancer mostly showed non-significant decreased associations with insulin use vs non-insulin use (drug exposure undefined) (Table 3). These studies did not distinguish between insulin analogues and human insulin. In contrast, most studies that compared insulin users with NIAD users (irrespective of the type of NIAD used) showed non-significant increased associations with risk of breast cancer. Only one study comparing insulin users versus non-insulin-users showed a statistically significant decreased risk of breast cancer (HR 0.86; 95 % CI 0.81, 0.91) in patients with type 2 diabetes [60]. However, we judge this study to be biased because the risk estimates were not adjusted for important risk factors for breast cancer and DM, immortal time bias might be present, and no data on duration of exposure were available. Exposure categories (insulin use-no insulin use and insulin use-NIAD only use) are hard to define and compare, because many patients with type 2 diabetes are using insulin (analogues) simultaneous with NIADs. Most studies that are included in this review investigated combined categories of exposure to insulin (analogues) and NIADs.

### Dose and duration effects in epidemiological studies

No significant differences were found between strata of duration and risk of breast 5 five years of any insulin treatment (HR 2.25; 95 % CI 0.72, 6.99) [62]. Among the glargine users, the study with the longest follow up comparing exposure of 4–7 years versus <4 years did not observe increased breast cancer risk [49]. Another study revealed that the risk of breast cancer increased in the first 3 years after the start of insulin glargine use, after which the risk of breast cancer remained at the same level [56]. Results of the effect of glargine dose on the occurrence of breast cancer [47, 49, 56, 58, 59, 64] produced inconsistent results (Additional file 1: Table S4). Some studies found significantly increased relative risks with increasing dose [56, 59, 64], while others did not [47, 49, 58, 59]; this seems partly dependent on the exposure definition. Only one of the studies investigating glargine dose used cumulative dose [47]. The results of one in vivo study in humans indicated that there is almost no glargine circulating in plasma regardless of the dose given. Plasma M1 concentration increased with increasing dose of glargine, but as was mentioned previously, M1 possesses low mitogenic signalling [70].

## Discussion

### Limitations of the studies and interpretation of the findings

#### In vitro studies

The large variation in published in vitro results can be explained by differences in study design. For example, the choice of cell line greatly affects the obtained results because the responsiveness to growth factors, like insulin and insulin analogues, may be different from one cell line to another. Based on the cell line characterisation (Additional file 1: Table S1), we showed there is a striking variation in receptor expression of the human cell lines used.

Different cell lines also have different expression of the relevant receptors involved in the insulin response. The MDA-MB-231 cell line has very low expression of IGF1R. Therefore, the increased mitogenic potential of glargine (due to enhanced IGF1R signalling) could not be detected in this cell line [28]. However, using the MCF7 cell line (which expresses very high levels of IGF1R) the increased mitogenic potential of this compound became evident [28]. Other cell lines with low or moderate expression levels of IGF1R are less suitable for a mitogenic evaluation of insulin analogues. In line with this, a recent study including four different breast cancer cell lines (MCF7, MDA-MB-157, MDA-MB-468 and T47D) found that mitogenicity of growth factors strongly depends on the cell line that was used [29]. However, the authors concluded that the INSR/IGF1R status was not the only explanatory factor. Therefore, we determined the expression of downstream signalling molecules (Fig 2). This illustrated that the poor responsiveness in the T47D and MDA-MB-468 cell lines upon glargine exposure [25, 29] may be explained by low expression of IRS1 (T47D) or IRS2 (MDA-MB-468), the first downstream targets of the INSR/IGF1R.

Besides INSR/IGF1R signalling other receptors also might have a role in insulin (analogue)-induced mitogenicity. Due to insulin-oestrogen receptor/progesterone receptor (ER/PR) crosstalk the IRS1 and subsequently the PI3K and MAPK signalling cascades can be upregulated resulting in enhanced proliferation [72]. This effect might contribute to the increased insulin (analogue) sensitivity of MCF7, T47D and ZR-75-1 compared to the triple-negative cell lines (MDA-MB-157, MDA-MB-231, MDA-MB-468 and MCF10A). Therefore, it is important to point out that primarily ER-positive or triple-negative breast cancer cell lines have been used in the included studies.

The majority of the mitogenicity studies used the MCF7 cell line [23–35]. It is desirable that future studies include different cell lines, so that cell-line-specific effects can be excluded. For translational reasons it is essential that protein expression (and especially receptor profiles) in benign human mammary gland tissues are quantified, only in that way we can determine which cell model has the highest clinical relevance.

Another important quality factor is the starvation method. For a proper effect of a specific stimulation it is essential that the target cells are deprived from other growth factors. Some studies did not starve their cells prior to the start of the assay [21, 25, 28, 33]; for short-term assays especially, this might have major consequences. Finally, the use of proper positive and negative controls is most important for a good quality experiment. Some studies [32, 33] did not include a positive control while others lack a negative control [23], thereby making it impossible to put the results in perspective. Furthermore, one study did include a positive control (IGF1) [24], but this compound did not show a positive effect, which questions the sensitivity of their experiments.

#### Animal studies

The type of animal model used plays a major role in the quality of animal studies. Generally, it is thought that rats are more sensitive in terms of carcinogenicity towards compounds and have a higher clinical relevance than mouse models [73]. But there are also major disadvantages, like higher costs and the lack of good humanized breast cancer rat models. Two studies that used rats have rather small group sizes, which obviously affects the power of their studies [37–39]. The doses that were used in the reviewed animal studies are quite comparable to each other and are all thought to be supra-physiological (i.e., over 50 times the human dose, based on nmol/kg). In one study a non-equimolar comparison was made between the different compounds, but doses had been chosen to induce an equi-pharmacological/metabolic response [40]. High mortality was observed in another study, probably due to hypoglycaemia, therefore the dose was lowered in a later phase of this study [39]. Surprisingly, other studies that used similar doses did not observe hypoglycaemia [37, 38, 40]. To verify the sensitivity of the models and techniques it is essential that the appropriate controls are included. Half of the included animal studies lacked proper controls. In our opinion both insulin and IGF1 (and ideally also AspB10) should always serve as controls to be able to put the obtained results into perspective.

#### Epidemiological studies

The epidemiological studies included in this review have many limitations and results are difficult to compare across studies because the exposure of interest and exposure comparison groups have been defined differently. For example, some studies compared glargine-only users with human-insulin-only users [64], while others compared glargine users with non-glargine-insulin users [66]. In this case, the comparator is a mix of several exposures, which may affect the conclusion about the effect of certain insulins (analogues). Some studies examined several definitions for the exposure of interest and indeed this resulted in slightly different effect estimates [57, 59]. Moreover, it is difficult to disentangle the effect of insulin and the role of NIADs because most diabetic patients treated with insulin have prescriptions of NIADs as well. However, it is important to do so, because some studies have shown anti-tumour effects of metformin, the most prescribed NIAD among patients with type 2 diabetes [74]. Of note, the quality of some of these metformin studies is doubtful as well.

Inclusion criteria differed largely among studies. For example, some studies included patients with only one insulin prescription while others included continuous users over a period of 6 months. More important, there was large variation in the definition of time of exposure. Some studies determined the use of different insulin types at baseline or during a fixed period (intention to treat), while others determined insulin exposure during follow up (time-dependently). This may lead to patients with only one specific insulin prescription during follow up being falsely classified as continuous users during the whole period. Cumulative exposure over time, censoring for discontinuation, or switching and latency period could affect the results. The uncertainty surrounding the extent to which a registered prescription dispensed for an insulin analogue reflects real life use of insulin analogues limits the ability to detect the true effect on the occurrence of breast cancer. Furthermore, studies variably included incident and prevalent users of insulin compromising estimates of association between the duration of use and breast cancer development.

Other methodological aspects that are important when interpreting the results of these studies are incorrect and too short an exposure time (maximum 3.8 years mean exposure time), reverse causation, confounding by indication, and residual confounding (Additional file 1: ESM 3). Most studies were based on type 2 DM, and/or did not specify the type of DM. Risk of bias was classified as low (for definition see Additional file 1: ESM 3) in only five studies [42, 46, 49, 62, 63], but the power of these studies was inadequate (Additional file 1: Table S5). Of these studies, only two considered breast cancer as a main outcome [49, 62]. Most risk estimates have wide CIs, due to lack of power of the study. Two of the three studies that found significantly different results were classified as having a high risk of bias [57, 60] or had lack of power [57, 64]. So far there is not a single very well-designed study to have investigated insulin treatment and breast cancer risk as the main outcome, with sufficient power. The included RCTs had limitations too, such as limited follow up (except for one RCT with a follow up of 6 years [42]), insufficient power, or cancer incidence as a secondary outcome [63, 75].

### All layers of evidence in perspective

Studies in humans are the gold standard for evaluating evidence of exposure and disease. The epidemiological studies reviewed varied in study design and exposure definition to too large an extent among different insulin analogues to evaluate their impact on breast cancer risk estimates. The risk estimates seemed not to be biased by important confounders, as adjusted and unadjusted risk estimates only differed slightly. However, unmeasured confounding may still be present. In addition, the upper limit of the 95 % CI of the pooled risk estimate of breast cancer among glargine users was 1.17. This strengthens our idea that if any, the increased risk of breast cancer due to currently used insulin (analogues) is likely to be very small.

A distinction should be made between studying tumour initiation or progression, though in the human setting it difficult to discern these because of potential lag time in the detection of cancer. The epidemiological studies investigated the incidence of primary breast tumours upon insulin treatment in DM patients. True tumour initiation in animal studies can only be investigated with long-term exposure in rodents, which are costly experiments. The animal xenograft models and in vitro studies of mammary tumour cell lines summarized here investigated tumour progression; e.g., by evaluation of cell proliferation or upregulation of mitogenic pathways. All together, the results of this systematic review suggest that insulin treatment might be involved in tumour promotion.

Another issue to be raised is that breast cancer is not one disease but consists of different subtypes, e.g., ER-positive or ER-negative cancer with different prognoses. The promotion of tumour cell growth upon insulin exposure may differ for different breast cancer subtypes. However, there are very limited human/epidemiological data from only two studies on the association of tumour subtypes and insulin (analogues) exposure among diabetic patients with breast cancer [49, 76]. More data are available on the prognosis of diabetic patients with breast cancer. It has been shown that overall mortality after breast cancer diagnosis is 25 to 50 % higher in diabetic women compared to their non-diabetic counterparts [45, 62, 77, 78], even after adjustment for tumour stage [77, 78]. However, whether this increased mortality is breast cancer-related or caused by comorbidities related to DM is not clear. Breast cancer in patients with DM is often diagnosed at an advanced stage compared to patients without DM [77–80]. But studies that investigated the association between breast cancer-specific mortality and diabetes have inconsistent results [45, 78, 80, 81]. Among patients with type 2 DM, insulin treatment is associated with a worse cancer outcome and increased all-cause mortality compared to metformin treatment [78, 82]. Only one study investigated the effect of cumulative dose and duration of insulin treatment on breast-cancer-specific survival, and found lower mortality from breast cancer [83].

## Conclusion

Based on the current epidemiological and animal data there is no compelling evidence that any clinically available insulin analogue, or human insulin increases breast cancer risk. However, animal data were limited and there is not a single very well-designed epidemiological study to have investigated insulin treatment and breast cancer risk as the main outcome, and with sufficient power. Large randomized clinical trials were negative for increased breast cancer risk with glargine, but longer follow up may be needed to detect delayed or smaller effects. In vitro studies have shown that only insulin AspB10 and glargine have increased mitogenic potential compared to regular human insulin in breast cancer cell lines. The relevance of this finding for the clinical situation is unknown because AspB10 is not used in humans and it has been shown that glargine is rapidly metabolized in vivo into M1 and M2, metabolites with low mitogenic potential. Evidence on the potential pathways involved in insulin-analogue-induced breast cancer mitogenesis is limited.

### Unanswered questions and future research

Except for insulin AspB10, which has never been available to humans, all insulin analogues are still marketed. Although, there is evidence from in vitro data that insulin glargine has increased mitogenic potential, so far, epidemiological studies have not shown evidence for an association between insulin (analogue) treatment and breast cancer risk in female diabetic patients. However, due to a relatively short follow up time in the epidemiological studies, it cannot be excluded that diabetic patients with pre-neoplastic lesions might be at higher risk of developing an invasive tumour when given a specific insulin treatment. Research on this topic is important but is still largely lacking. Therefore, we are awaiting the results of ongoing efforts to pool multiple large national databases from different countries to perform a retrospective observational study in humans with a proper design, enough patients and long follow up. Additionally, further research into the aetiology of insulin and breast cancer development is important.

## Additional file

Additional file 1:
**ESM 1.** Search strategy for each database, study selection and results. ESM 2. Characterization of cells lines. Additional materials and methods section. ESM 3. Method for quality evaluation of epidemiological studies. ESM 4. Description of the included studies. **Table S1.** Protein and gene expression of hormone receptors for in vitro human mammary cell lines included. **Table S2.** Description of epidemiological studies included in the systematic review. **Table S3. a** Characteristics of the case control studies included in the systematic review. **b** Characteristics of the cohort studies included in the systematic review. **c** Characteristics of the randomized clinical trials included in the systematic review. **Table S4.** Relative risk estimations for breast cancer among different duration and dose categories within insulin treatment groups. **Table S5.** Quality evaluation of the epidemiological studies included in the systematic review*. **Figure S1.** Forest plot of breast cancer risk among insulin (analogue) users stratified by treatment group and type of effect estimate. Different exposure comparisons within one study are indicated by a, b, c. The exposure comparison can be found in Table 3 and Additional file 1: Table S2. (PDF 457 kb)

## Abbreviations

AKT: protein kinase B
BrdU: 5-Bromo-2’-deoxyuridine
BSA: bovine serum albumin
DM: diabetes mellitus
DMEM: Dulbecco’s modified Eagle’s medium
EGFR: epidermal growth factor receptor
EMT: Epithelial to mesenchymal Transition
ER: oestrogen receptor
ERK: extracellular signal regulated kinase
FACS: fluorescence-activated cell sorting
FBS: foetal bovine serum
FCS: foetal calf serum
GAPDH: glyceraldehyde-3-phosphate dehydrogenase
HER2: human epidermal growth factor receptor 2
HI: human insulin
HR: hazard ratio
IGF1R: insulin-like growth factor 1 receptor
INSR: insulin receptor
IRR: incidence rate ratio
IU: international unit
MAPK: mitogen activated protein kinase
mTOR: mechanistic target of rapamycin
NIAD: non-insulin anti-diabetic drug
OR: odds ratio
PI3K: phosphatidylinositol-4,5-bisphosphate 3-kinase
PR: progesterone receptor
PRISMA: preferred reporting items for systematic reviews and meta-analyses
PTEN: phosphatase and tensin homolog
RCT: randomized controlled trial
RR: relative risk
Sig: significance
TZD: thiazolidinedione
WB: Western Blot
