# Supplementary material for: Treatment with insulin (analogues) and breast cancer risk in diabetics; a systematic review and meta-analysis of in vitro, animal and human evidence
Source: Breast Cancer Res. 2015 Aug 5;17(1):100. doi: 10.1186/s13058-015-0611-2 (PMC4531810; doi:10.1186/s13058-015-0611-2)
Supplement: Additional file 1: — ESM 1. Search strategy for each database, study selection and results. ESM 2. Characterization of cells lines. Additional materials and methods section. ESM 3. Method for quality evaluation of epidemiological studies. ESM 4. Description of the included studies. Table S1. Protein and gene expression of hormone receptors for in vitro human mammary cell lines included. Table S2. Description of epidemiological studies included in the systematic review. Table S3. a Characteristics of the case control studies included in the systematic review. b Characteristics of the cohort studies included in the systematic review. c Characteristics of the randomized clinical trials included in the systematic review. Table S4. Relative risk estimations for breast cancer among different duration and dose categories within insulin treatment groups. Table S5. Quality evaluation of the epidemiological studies included in the systematic review*. Figure S1. Forest plot of breast cancer risk among insulin (analogue) users stratified by treatment group and type of effect estimate. Different exposure comparisons within one study are indicated by a, b, c. The exposure comparison can be found in Table 3 and Additional file 1: Table S2. (PDF 457 kb) [file 13058_2015_611_MOESM1_ESM.pdf]

Electronic supplementary material ‘Treatment with insulin (analogues) and breast cancer risk in diabetics; a review of in vitro, animal and human evidence‘

ESM 1. Search strategy for each database, study selection and results

Search strategy and study selection

Online literatures searches have been updated up to July 28th 2014. Subject headings and Mesh terms were used for the search depending on the database used. We also searched in references lists of the identified reviews for papers we missed. There were no restrictions on publication date or publication status. Articles in Dutch or English were included. Two reviewers (HKB, BtB), developed and performed the search strategy for each database; duplicate references were removed (figure 1). Both reviewers independently screened title and abstract of the records for inclusion. BtB assessed the full text records of in vitro and animal studies, HKB of epidemiological and cohort studies for inclusion in the review. Reasons for exclusion were discussed.

Search terms

Web of Science
TS=("insulin analo*" OR "insulin derivative*" OR "insulin homolo*" OR glargine OR LANTUS OR degludec OR tresiba OR NPH OR lispro OR humalog OR detemir OR levemir OR glulisine OR apidra OR aspart OR novolog OR AspB10 OR X10 OR “insulin treatment” OR “diabetes treatment” OR “insulin therapy” OR “diabetes therapy”) AND TS=("mammary gland" OR "breast neoplas*" OR "mammary tumor" OR "mammary cancer" OR "breast cancer " OR “breast carcinoma” OR malignan* OR carcinog* OR mitoge*)
# of articles: 587

Medline (PubMed)
((("insulin analogue" OR “insulin analogues" OR “insulin analog” OR “insulin analogs” OR "insulin derivative" OR "insulin derivatives" OR "insulin homologue" OR "insulin homologues" OR glargine OR LANTUS OR degludec OR tresiba OR NPH OR lispro OR humalog OR detemir OR levemir OR glulisine OR apidra OR aspart OR novolog OR AspB10 OR X10 OR “insulin treatment” OR “diabetes treatment” OR “insulin therapy” OR “diabetes therapy”)[Title/Abstract]) OR "Insulin/analogs and derivatives"[MeSH]) AND (("mammary gland" OR "breast neoplasia" OR "mammary tumor" OR "mammary cancer" OR "breast cancer" OR "breast carcinoma" OR malignancy OR carcinogen OR carcinogenic OR mitogen OR mitogenic[Title/Abstract]) OR "Breast Neoplasms”[MeSH]))
# of articles: 1212

Embase
insulin derivative/ or insulin aspart/ or insulin aspart plus insulin degludec/ or insulin degludec/ or insulin detemir/ or insulin glargine/ or insulin glulisine/ or insulin lispro/ or long acting insulin/ or short acting insulin/ AND breast cancer/ or breast tumor/ or breast carcinogenesis/
# of articles: 240

ESM 2. Characterization of cells lines

Cell line selection and culturing

Cell lines that were studied in the *in vitro* experiments are; MCF7, T47D, MDA-MB-157, MDA-MB-231, MDA-MB-468, Hs578T, ZR-75-1 and MCF10A. These cell lines are often used in other *in vitro* studies included in this systematic review. All cell lines were obtained from ATCC (Manassas, VA, USA) and were kindly provided to us by John A. Foekens and John W.M. Martens (Erasmus University Medical Center, Rotterdam, The Netherlands).
Cells were seeded in a 6-well format at a confluence of 60% in RPMI 1640 (Gibco, Invitrogen, Carlsbad, CA, USA) supplemented with 10% fetal bovine serum (FBS) and 100 U/mL penicillin-steptomycin (Invitrogen). Plates were incubated for 30 hours at 37˚C and 5% CO_2 followed by cell lysis.

Antibodies and reagents

Antibodies against rabbit anti-phospho-IGF1Rβ (tyr1135/1136)/phospho-IRβ (Tyr1150/1151), anti-Akt, anti-phospho-Akt (Ser473), anti-Erk, anti-phospho-Erk (Thr202,Tyr204), anti-HER2 (Cell Signaling Technology, Danvers, MA, USA), mouse anti-IGF1Rβ, anti-β-Actin, anti-GAPDH and rabbit anti-IRβ, anti-EGFR, anti-ER-α, anti-IRS-1, anti-IRS-2, (Santa Cruz Biotechnology, Santa Cruz, CA, USA) and mouse anti-α-tubulin and rat anti-E-cadherin (Sigma-aldrich, St. Louis, MO, USA) and mouse anti-N-cadherin (BD translaboratories) were commercially purchased. Conjugated secondary antibodies included anti-mouse horseradish peroxidase (HRP), anti-rabbit HRP, anti-rat HRP, anti-goat HRP and Cy-5 conjugated anti-mouse have been purchased from Jackson (Jackson ImmunoResearch, West Grove, PA, USA).

Western blot analysis

The cell lysis, protein quantification and western blot analysis was performed as previously described by Li et al [1]. 40 µg of total protein was loaded per lane. For the tubulin, Actin and GAPDH blots, Cy-5 conjugated secondary antibodies were used which were visualized using a Typhoon 9400 imager. HRP conjugated secondary antibodies have been used for all the other proteins. These blots have been exposed to Pierce® ECL Western blotting substrate (Thermo Scientific, Rockford, IL, USA). Proteins were visualized by bringing the membranes in contact with an X-ray film (GE Healthcare, Little Chalfont, England). The film was developed with a Kodak X-omat 1000 processor. All bands have been

quantified using ImageJ software (ImageJ, 1.43u). To correct for loading perturbations all bands have been divided by the tubulin levels of that specific blot. ZR-75-1 cell line showed basal protein expression levels of all of the receptors. Therefore, the protein expression levels of all receptors have been normalized against the levels of ZR-75-1.

Gene expression analysis

For the gene expression analysis we a used RNA normalized micro-array data from the Sanger Institute (http://cancer.sanger.ac.uk/cell_lines/download). This dataset has ArrayExpress accession number E-MTAB-3610. In a gene wise manner we expressed these values as fold changes compared to the expression levels of ZR-75-1, as we did for the protein expression analysis.

ESM 3. Method for quality evaluation of epidemiological studies

After definition of the criteria, the epidemiological studies were evaluated for study quality by two reviewers (HKB, OK). Studies differ in methodological aspects. We focused on potential selection bias, information bias, confounding bias and lack of power on the basis of information presented in the publications. Risk of bias is summarized in low, moderate and high based on a (subjective) qualitative evaluation of selection, information and confounding bias (ESM 11). These variables that were used to determine risk of bias and lack of power are presented in the ESM7-9 and table 3 respectively.

Selection bias: For the follow up studies we first evaluated the selection of the index and control groups. We evaluated at baseline whether the cancer risk was already substantially different in both groups in a way the adjustment for difference in prognosis is not possible. Secondly, we evaluated loss-to-follow up, especially evaluating whether the loss-to-follow up was different in the index and control group and related to cancer/survival risk. Within the case-control studies we evaluated selection bias by evaluating whether the cases and controls came from the same population. If cases were not matched to controls on calendar time and potential exposure time, we considered if time window bias could be present.

Information bias: To evaluate whether exposure could have been misclassified we determined if exposure was measure cumulative over time, if investigators censored for switching or discontinuation of insulin treatment and whether a latency time was included. The variables data source exposure, time of exposure definition, the duration of exposure to insulin, prevalent/incident user and latency period were used to determine the above mentioned criteria. If studies did not include a latency period this could have led to breast cancer diagnosis, which was not due to the exposure of interest. This might have resulted in misclassification of the exposure-outcome relation. Studies with an intention to treat approach were indicative for risk of bias, as it assumes that the effects of exposure would continue beyond the exposure period. For the studies that reported the cumulative exposure, immortal time bias was considered. Immortal time bias was apparent if follow up (py/exposure of interest) includes unexposed time. Unknown exposure time before cohort entry in prevalent user cohort, was considered to lead to

information bias as well. It is known that one prescription of insulin is a good predictor for actual insulin analogue use of a diabetic patient. This have been proven for patients with diabetes type 1 [2], therefore we did not take exposure definition (minimum number of prescriptions to be defined as exposed) into account in this quality evaluation.

Confounding: To evaluate the potential bias due to confounding factors, we evaluated whether the effect estimations were matched or adjusted for the following variables: age, BMI, DM duration, other DM medication than medication of interest and physical activity. Also important risk factors for breast cancer were taken into account, like family history of BC, parity, age at menarche, age at first birth, menopausal status, HRT use and anti-contraceptive pill use. All variables that were not adjusted for are listed in ESM11.

Lack of power: The number of exposed patients to be studied to identify a relative breast cancer risk of 1.2 with 80% power, α=0.05 was calculated for cohort and case control. Cut off values of the minimum required number of exposed patients were used to evaluate if the studies included in the review had enough power. In addition, the number of breast cancer cases were taken into account, e.g. if a study includes a large population but follow up is short, the number of cases can still be small. For the cohort studies power was calculated using the methods described by Rothman [3] and Miettinen [4]. Cumulative breast cancer incidence over 10 years in Europe was calculated to estimate the risk in the unexposed patients (incidence rate per 100,000: 94.2) [5]. It was assumed that the ratio of unexposed versus exposed patients was 2:1 respectively. Based on these numbers our estimation was that the total required number of patients exposed to the insulin analogue of interest was 35,000 and 70,000 patients exposed to the reference compound. For case-control studies power was calculated using Power and Sample Size Program version 3.1.2. It was assumed that 1 cases was matched to 4 matched controls and that the probability of exposure to insulin among controls was 0.55%. Studies were powered to detect an OR of at least 1.2 based on recruitment of 1000 cases and 4000 controls.

Besides the type of bias that are included in the quality evaluation of the studies, other aspects are also important to take into account while interpreting the results of these studies. These methodological aspects have not been discusses per study, as some of these are applicable for most of the studies. First of all, incorrect definition of exposure time can lead to information bias. The longest duration of cumulative exposure was 3.5 years, while carcinogens have long latency periods. Secondly, studies may suffer from reverse causality. It might be due to subclinical phase of breast cancer that the need for insulin treatment changes and therefore it seems that insulin causes cancer while actually this is affected by the undetected breast cancer itself. Thirdly, studies may suffer from confounding by indication; subjects who use insulin are more likely to developing breast cancer due to other factors. Breast cancer incidence might differ between different diabetic medication even if the medication itself have not such an effect. There might also be systematic differences in characteristics between treatment groups. All cohort studies, except for one[6] were not matched on patient characteristics, which results in a lack of comparability and most likely residual confounding. Additionally, some studies included patients with DM1 and DM2. Most studies that only included DM2 patients, derived DM type based on the age at onset and cut offs were different across the studies. Furthermore, it is hard to distinguish between the role of diabetes itself in the potential carcinogenic effect and the role of insulin analogues. This might have biased the results. Randomized controlled trials are

free of confounding (by indication), but the trials that were included [7-10][6-9][7-10][7-10][7-10] had other limitations, such as short follow up, a lack of power and in 2 of the studies, the outcome of interest was a secondary objective. Therefore we cannot compare these results.

ESM 4. Description of the included studies

In vitro studies

Study characteristics of the in vitro studies are summarized in table 1. Seven different human breast cancer cell lines and one immortalized cell line were used. Protein expression of hormone receptors INSR, IGF1R, ER, PR, HER2 and EGFR and some downstream signalling proteins for each cell line are provided in figure 3 and table 2.

A total of 14 different assays are described. These assays have different readouts and therefore the conclusions that can be drawn are different. Proliferation assays (MTT, [H]Thymidine incorporation, Brdu incorporation, SRB, DNA measurement, Cristal violet cell staining, ki67 or Cell counting) will shed light on the direct mitogenic potential of the compounds, whereas with functional assays (colony forming assay, collagen invasion assay, Western blotting, FACS or Bret-PIP3)) a more specific question can be addressed (e.g. ability to invade or the involvement of a particular protein in a specific process).The experimental procedures varied significantly as well, e.g. the exposure time ranged from 5 min to 5 days.

Animal studies

Descriptions of the animal studies can be found in table 2. The number of relevant animal studies was very limited and the set-up varied largely.

Human studies

Four randomized clinical trials (RCT), 5 case-control studies (2 nested case-control studies) and 20 cohort studies were included. Twelve studies investigated the effect of any exposure to exogenous insulin on the incidence of breast cancer; Nineteen studies investigated different types of insulin analogues. For most insulin analogues very few studies were published, except for long acting insulin glargine (figure 1). Descriptions and characteristics of these studies are presented in ESM 6-9.

The status and definition of diabetes, and variables that relate to insulin exposure vary among studies. Seventeen studies restricted the study population to patients with DMT2 only, though the majority of patient in the other studies were also DMT2. Fifteen studies included only incident insulin users, i.e., patients who received their first insulin prescription during the study period. Total follow up ranged from 1.9 to 7.1 years, and mean duration of glargine treatment ranged from 0.9 to 3.5 years. Latency periods varied from 3 to 36 months.

Only two in vivo studies in humans have been performed. One study determined plasma levels of insulin glargine and its metabolites M1 and M2 after glargine injection in patients with type 1 DM. The other study investigated clinical and breast tumour characteristics of patients with diabetes treated with glargine or other insulin analogues.

References

1. Li J, Volpe DA, Wang Y, Zhang W, Bode C, Owen A, Hidalgo IJ: **Use of transporter knockdown Caco-2 cells to investigate the in vitro efflux of statin drugs**. *Drug Metab Dispos* 2011, **39**(7):1196-1202.
2. Rawshani A, Landin-Olsson M, Svensson AM, Nystrom L, Arnqvist HJ, Bolinder J, Gudbjornsdottir S: **The incidence of diabetes among 0-34 year olds in Sweden: new data and better methods**. *Diabetologia* 2014, **57**(7):1375-1381.
3. Rothman KJ, Boice JD: **Epidemiologic Analysis with a Programmable Calculator**. *NIH* 1979, **Publication No. 79-1649**.
4. Miettinen OS: **Individual matching in the case of all or noneresponses**. *Biometrics* 1969, **25**:339-354.
5. Ferlay J, Steliarova-Foucher E, Lortet-Tieulent J, Rosso S, Coebergh JW, Comber H, Forman D, Bray F: **Cancer incidence and mortality patterns in Europe: estimates for 40 countries in 2012**. *Eur J Cancer* 2013, **49**(6):1374-1403.
6. Suissa S, Azoulay L, Dell'Aniello S, Evans M, Vora J, Pollak M: **Long-term effects of insulin glargine on the risk of breast cancer**. *Diabetologia* 2011, **54**(9):2254-2262.
7. Dejgaard A, Lynggaard H, Rastam J, Krogsgaard Thomsen M: **No evidence of increased risk of malignancies in patients with diabetes treated with insulin detemir: A meta-analysis**. *Diabetologia* 2009, **52**(12):2507-2512.
8. Gerstein HC, Bosch J, Dagenais GR, Diaz R, Jung H, Maggioni AP, Pogue J, Probstfield J, Ramachandran A, Riddle MC *et al*: **Basal insulin and cardiovascular and other outcomes in dysglycemia**. *N Engl J Med* 2012, **367**(4):319-328.
9. Home PD, Lagarenne P: **Combined randomised controlled trial experience of malignancies in studies using insulin glargine**. *Diabetologia* 2009, **52**(12):2499-2506.
10. Rosenstock J, Fonseca V, McGill JB, Riddle M, Halle JP, Hramiak I, Johnston P, Davis M: **Similar risk of malignancy with insulin glargine and neutral protamine Hagedorn (NPH) insulin in patients with type 2 diabetes: Findings from a 5 year randomised, open-label study**. *Diabetologia* 2009, **52**(9):1971-1973.

ESM Table 1. Protein and gene expression of hormone receptors for *in vitro* human mammary cell lines included

Cell line	Origin/type of cells	INSR		IGF1R		INSR:IGF1R ratio		ER		PR		HER2		EGFR	
		P.E.	G.E.	P.E.	G.E.	P.E.	G.E.	P.E.	G.E.	P.E.	G.E.	P.E.	G.E.	P.E.	G.E.
**MCF7**	Adenocarcinoma/epithelial	1.19	0.76	7.48	2.41	1: 6.3	1:3.2	1.71	1.15	0.11	8.31	0.04	0.11	0.20	0.99
**T47D**	Ductal Carcinoma/Epithelial	0.47	0.38	2.43	1.23	1: 5.2	1:3.2	1.03	0.80	10.68	12.11	0.54	0.13	0.30	1.08
**MDA-MB-157**	Medulallary carcinoma/Epithelial	1.19	1.20	0.11	0.77	1: 0.1	1:0.6	0.00	0.12	0.02	0.00	0.02	0.12	10.73	1.03
**MDA-MB-231**	Adenocarcinoma/Epithelial	1.58	0.61	0.97	0.20	1: 0.6	1:0.3	0.00	0.00	0.03	0.08	0.01	0.00	10.59	1.50
**MDA-MB-468**	Adenocarcinoma/Epithelial	1.54	0.76	0.48	0.00	1: 0.3	NA	0.00	0.03	0.03	0.43	0.00	0.03	53.06	2.61
**Hs578T**	Carcinoma/Fibroblast	0.00	0.00	0.01	1.16	1: 4.7	NA	0.00	0.04	0.02	0.16	0.00	0.02	20.29	1.38
**ZR-75-1**	Ductal Carcinoma/Epithelial	1.00	1.00	1.00	1.00	1: 1.0	1:1.0	1.00	1.00	1.00	1.00	1.00	1.00	1.00	1.00
**MCF10A**	Mammary gland (benign) /Epithelial	1.22		1.48		1: 1.2		0.00		0.01		0.43		4.56	

Abbreviations: P.E.= the quantified protein expression levels based on the Westernblot analysis (fig 2). G.E.= the quantified gene expression levels of the corresponding cell lines based on the Micro Array data of the Sanger Institute.

ESM Table 2. Description of epidemiological studies included in the systematic review

Author, Year, Country of study	Study design	Source population	Data source population	Diabetes type and definition	Indicator for the exposure comparison ***	Exposure comparison (n)**<br><br>Exposure group	Exposure comparison (n)**<br><br>Reference	Age
Bodmer et al, 2010 [41] UK	Nested case-control	Nationwide	General Practice Research Database (GPRD)	T2DM: diagnosed > 30 years	a	Insulin users (43/131)	No insulin users (262/1,022)	30-79
Cleveland et al, 2012 [45] USA	Case-control	Population (Nassau and Suffolk counties of Long Island)	Long Island Breast Cancer Study Project (LIBCSP)	T2DM: diagnosed ≥ 30 years		Insulin users (20/16)	No insulin users (50/49)	Mean: DM 63.6 non-DM 57.4
Grimaldi-Bensouda et al, 2013 [49] UK, Canada, France	Case-control	France: nationwide UK: England,Scotland Canada: Quebec, Ontario, and New-Brunswick	Oncology clinics (medical records)	T1DM/T2DM; NR	a	Glargine users (78/287)	Non-glargine users (697/2,763*)	≥ 18
					b	Glargine users (74/203)	Non-glargine insulin users (70/207)	
					c	Glargine users (NR)	Human insulin users (NR)	
					d	Glargine users (NR)	Aspartat users (NR)	
					e	Glargine users (NR)	Lispro users (NR)	
					f	Aspart users (54/241)	Non-aspart users (721/2,809*)	
					g	Lispro users	Non-lispro users	

						(46/133)	(729/2,917*)	
					h	Human insulin users (59/260)	Non-human insulin users (716/2,790*)	
Koro et al, 2007 [54] USA	Nested case-control	NR (covers 9 census region; 30 different healthcare plans; 38 million patients)	Insurance database (Integrated Healthcare Information Services (IHCIS)	T2DM: ICD-9 code 250.x.	a	Insulin and NIAD users (13/52)	TZD users (83/449)	≥ 18
					b	Insulin only users (9/62)	TZD users (83/449)	
Mannucci et al, 2010 [58] Italy	Nested case-control	Florence	Diabetes cohort	T2DM; clinical diagnoses	a	Glargine users (NR)	Non-glargine insulin users (NR)	Mean: cases 68.9 ± 9.9, controls 68.0 ± 10.0
Carstensen et al, 2012 [43] Denmark	Cohort	Nationwide	Diabetes register (National Danish Diabetes Register)	T2DM: diagnosed 35 years		Insulin users (NR)	No insulin users (NR)	all
Chang et al, 2011 [44] Taiwan	Cohort	Nationwide	Insurance database (Taiwan's National Health Insurance (TNHI) claims database)	T2DM; T1DM excluded: ICD-9 code 250.x1 or 250.x3		Glargine users, not using int-/long-acting HI (4,566)	Non-glargine int/long-acting HI users (23,377)	≥ 18
Colhoun et al, 2009 [5] Scotland	Cohort	Nationwide	Clinical diabetes database (Scottish Care Information-Diabetes Collaboration (SCI-DC))	T2DM: diagnosed ≥ 35 years	a	Glargine plus non-glargine insulin users (NR)	Non-glargine insulin users (NR)	adults
					b	Glargine only users (NR)	Non-glargine insulin users (NR)	
Currie et al, 2009 [6] UK	Cohort	Nationwide	General practice database (The Health Information Network (THIN))	T2DM: diagnoses > 40 years	a	Insulin users (4,432)	Metformin only (13,834)	Mean: 63.7 ± 12.9
					b	Glargine users (959)	Non-glargine insulin users	

							(2,314)	
Fagot et al, 2013 [47] France	Cohort	Nationwide	Insurance database (French National Health Insurance Information system (SNIIRAM))	T2DM: ≥3 NIAD prescriptions in calendar year before exposure to insulin	a	Glargine users (25,298)	Other int-/long-acting insulin only users (8,687)	40-79
					b	Determir users (8,302)	Other int-/long-acting insulin only users (25,683)	
					c	Basal human insulin users (3,401)	Other int-/long-acting insulin only users (30,584)	
Ferrara et al, 2011 [48] USA	Cohort	Northern California	Diabetes register (Kaiser Permanente Northern California Diabetes Registry (KPNC))	NR; diabetes related records from several sources		Insulin users (51,511)	No insulin users (200,956)	≥ 40
Gu et al, 2013 [50] China	Cohort	Shanghai	Shanghai Diabetes Register (SDR) database	T2DM; NR (from DM register)		Human insulin users (1,765)	No insulin users (2,340)	> 30
Habel et al, 2013 [51] USA	Cohort	Northern and Southern California	Health plan register (Kaiser Permanente Northern and Southern California (KPNC and KPSC))	T1DM/T2DM; Diabetes related records from several sources	a	Glargine users (2,869)	NPH insulin users (19,591)	≥ 18
					b	Glargine only users (NR)	NPH insulin users (19,591)	
					c	Glargine and NPH insulin users (NR)	NPH insulin users (19,591)	
Hsieh et al, 2012 [53] Taiwan	Cohort	Random sample of nationwide database	Insurance database (Taiwan's National Health Insurance (NHI) claims database)	T2DM: ICD-9 code 250.x0 or 250.x2		Insulin only users (338)	Metformin only users (2,048)	Mean: 61.4 ± 13.2
Kostev, 2012 [55] Germany	Cohort	NR (covers 20 million patients in Germany)	Research database with data from general practitioners and	T2DM; NR	a	Glargine users (4,727)	NPH insulin users (4,206)	Mean: 67.5 ± 11.2

(Letter)			clinical specialists (IMS Disease Analyzer)					
					b	Determir users (789)	NPH insulin users (4,206)	
Lind et al, 2012 [56] Sweden	Cohort	NR (17 hospitals in Sweden)	Clinical diabetes database (Diab-base)	T1DM(42%)/T2DM/unspecified; NR (from DM register)	a	Glargine users (2,014)	Non-glargine users (5,928)	13-97
Ljung et al, 2011 [57] Sweden	Cohort	Nationwide	Prescription database (combination of Prescribed Drug Register	T1DM: diagnosed < 30 years and T2DM: diagnosed > 30 years	a	Glargine plus non-glargine insulin users (8.889)	Non-glargine insulin users (38,152)	35-84
					b	Glargine only users (2,697)	Non-glargine insulin users (38,152)	
Morden et al, 2011 [59] USA	Cohort	Nationwide	Insurance database (Medicare)	T2DM; ICD-9 code 250.x0 or 250.x2	a	Glargine plus non-glargine insulin users (10,375)	Non-glargine insulin users (34,789)	≥ 68
					b	Glargine only users (10,857)	Non-glargine insulin users (34,789)	
Neumann et al, 2012 [60] France	Cohort	Nationwide	Insurance database (French National Health Insurance Information system (SNIIRAM)	T2DM: NIAD prescriptions in calendar year before exposure to insulin		Insulin users (179,618*)	No insulin users (491,892*)	40-79
Onitilo et al, 2014 [61] USA	Cohort	North-central Wisconsin	Marshfield Clinic electronic medical records (EMR)	T2DM; ICD-9 code 250.x0 or 250.x2		Insulin users (1,377*)	No insulin users, hba1c >7% (3,153*)	≥ 30
Redaniel et al, 2012 [62] UK	Cohort	Nationwide	General Practise Research Database (GPRD)	T2DM: diagnosed ≥ 35 years	a	Insulin and NIAD users (2,127)	Sulfonylurea only users (4,815)	> 35
					b	Insulin only users (434)	Sulfonylurea only users (4,815)	
Ruiter et al, 2012 [64] Netherland	Cohort	Pharmo database from community pharmacies in the	Prescription database (PHARMO)	T2DM; T1DM excluded: patient using only insulin	a	Glargine only users (1,888)	Human insulin only users (5,093)	≥ 18

s		Netherlands (covers 2.5 million individuals)						
					b	Non-glargine insulin users (3,101)	Human insulin only users (5,093)	
Sturmer et al, 2013 [65] USA	Cohort	NR, US citizens enrolled in a health plan (covers >76 million inidividuals; 295 000 physicians; 185 000 clinical facilities)	Health plan registry (Inovalon Medical Outcomes Research for Effectiveness and Economics Registry (MORE))	T1DM/T2DM; ICD-9 code 250.xx	a	Glargine users (22,936)	NPH users (5,536)	≥ 18
Suissa et al, 2011 [66] UK	Matched cohort ****	Nationwide	General Practise Research Database (GPRD)	T2DM: diagnosed ≥ 40 years	a	Glargine users (1,604)	Non-glargine insulin users (3,086)	≥ 40
Vallarino et al, 2013 [67] USA	Cohort	NR, US citizens enrolled a healthcare insurance plans (covers 47 million individuals)	United healthcare insurance plan database (i3 InVision Data Mart)	T2DM; ICD-9 code 250.x0 or 250.x2		Pioglitzone users, not using insulin (15,589)	Insulin users, not using pioglitazone (8,444)	≥ 45
Bordeleau et al, 2014 [42] Canada	RCT	International multicentre study (40 countries)	Clinical sites participating in the ORIGIN trial	Impaired fasting glucose, impaired glucose tolerance, early T2DM; clinical diagnosis		Glargine users (6,264)	Standard care, not using glargine (6,273)	≥ 50
Dejgaard et al, 2009 [46] Denmark	Several RCTs	NR, participants of 21 Novo Nordisk-sponsored RCTs	Individual patient data (IPD) from Novo Nordisk sponsored trials	T1DM (9 studies)/T2DM (11 studies); NR	a	Determir users (3,983)	NPH users (2,661)	adults
					b	Determir users (1,219)	Glargine users (830)	
Home and Lagarenne 2009 [52] UK, USA	Several RCTs	NR, participants of 31 RCTs registered at sanofis-aventis	Pharmacovigilance database (sanofi-aventis)	T1DM (12 studies)/T2DM (19 studies); NR		Glargine users (5,657)	Any anti-diabetic drug, NPH in 20 studies (5,223)	all
Rosenstock	RCT	Multicentre study in	Medical centres	T2DM; diagnosed for		Glargine users	NPH users (503)	30-70

et al, 2009 [63] USA, Canada	USA and Canada	participating in RCT	≥ 1 year	(514)

*Calculated using data provided (if not indicated directly taken from table in paper), ** In case-control studies, n reflects cases/controls, *** Different exposure comparisons within one study are indicated by a,b,c etc. , **** Matched on birth year; calender time. Abbreviations: NR= not reported, T1DM= type 1 diabetes mellitus, T2DM= type 2 diabetes mellitus, BC= breast cancer, NIAD=non-insulin anti-diabetic drug

ESM Table 3a. Characteristics of the case control studies included in the systematic review

Author, Year	Study period	Data source controls*	Matching variables*	Data source exposure**	Data source outcome**	Exposure definition ****	Time of exposure definition**	Duration of exposure prior to index date**	Latency period**	Exclusion of patients with a history of cancer	Covariates***
Bodmer et al, 2010 [41]	1994-2005	General Practice Research Database (GPRD)	Age; general practise; index date	General Practice Research Database (GPRD); prescribed	General Practice Research Database (GPRD)	≥ 1 oral drug prescription	During study period: in mutually exclusive treatment groups	mean: NR, # prescriptions in categories (Sup T4)	0	Yes, any cancer	BMI; hba1c; DM duration; smoking; acarbose; oestrogen; other DM medication ******
Cleveland et al, 2012 [45]	Life time exposure, BC diagnosis 1996-1997	Long Island Breast Cancer Study Project (LIBCSP). Controls were female residents from Nassau and Suffolk	Frequency matched by 5-years age groups	Interview	Physician and medical records	≥ 3 months consecutive treatment with ADD	Recalling past diabetes medication by a interview at study inclusion	NR	0	Yes, breast cancer	BMI; menopausal status; race; other DM medication ******
Grimaldi-Bensouda et al, 2013 [49]	2000-2009	General practitioners network; Pharmaco-epidemiologic General Research eXtension program (medical	Age; type of DM; country/region; date of recruitment; referral to diabetologist	Interview, validated by prescriptions from GP records	Pathology and computerized oncology records	≥ 3 months treatment with insulin	During study period: for each ADD exposure (yes/no) was defined	Mean for glargine (years): 3.2 ± 2.0 in whole study population	3	Yes, breast cancer	Age; BMI; DM duration; breast cancer risk score (many variables); comorbidities; annual number of physician visits; oral ADD use; past-insulin use; other insulin use; other

		records)									medication use
Koro et al, 2007 [54]	1997-2004	Insurance database (IHCIS)	Age; sex; index date; lenght of follow-up in the database	Insurance database (IHCIS); claims	Insurance database (IHCIS)	≥ 1 ADD prescription	During study period: for each ADD exposure (ever/never) was defined. Mutually exclusive treatment groups were made	NR	0	Yes, breast cancer	Age
Mannucci et al, 2010****** [58]	1998-2008	Diabetes cohort	Age; sex; BMI; lenght of follow-up	Clinical records; prescriptions	Hospital admission (Regional Hospital Discharge System) or death register (Mortality register of Tuscany)	≥ 1 insulin prescription	During study period: for each insulin type duration and mean daily dose of treatment was calculated	Median for glargine (years): 1.67 (0.8-2.3) in cases, 1.2 (0.4-2.2) in controls	12	Yes, any cancer	Comorbidity; metformin; total insulin dose; dose per insulin type; proportion of subjects with MDD ≥0.3 IU/kg/day per insulin type

* used to evaluate potential selection bias, ** used to evaluate potential information bias, *** used to evaluate potential confounding bias, **** minimum number of prescriptions during a specified period, ***** incident users, ****** other covariates were assessed but not included in the final model as they had no impact on the risk estimate. Abbreviations: NR= not reported, DM= diabetes mellitus, BC= breast cancer, ADD= anti-diabetic drugs

ESM Table 3b. Characteristics of the cohort studies included in the systematic review

Author, Year	Study period	Data source exposure**	Data source outcome**	Prevalent / incident user**	Exposure definition ****	Time of exposure definition**	Mean duration of exposure (years)**	Latency period**	Exclusion of patients with a history of cancer	Covariates***
Carstensen et al, 2012 [43]	1995-2009	Diabetes register or prescription database	Danisch Cancer Registry	Incident	≥ 2 insulin prescriptions	During follow-up (intervals): exposure status and duration were updated	NR	1	Yes, any cancer	Age; date of birth; sex; calender time
Chang et al, 2011 [44]	2004-2007	Insurance database (TNHI); claims	Insurance database (TNHI)	Incident	≥ 1 insulin prescription	During follow-up: exclusive users during whole follow-up period	Glargine: 1.4<br>HI: 2.0	0	Yes, any cancer	Age; DM-related complications; comorbidities; health service utilization; outpatient visits diabetes and non-diabetes; physician characteristics; statins; aspirin; initiation year insulin; dose of fast-acting insulin
Colhoun et al, 2009 [5]	2002/3-2005	Clinical diabetes database (SCI-DC))	Cancer register (Scottish Morbidity Record) and death register (General Registrar's Office for Scotland)	Incident	≥ 1 insulin prescription during 4 months period	During fixed period (4 months), follow-up starts after this period	NR	4	No; exclusion of patients with prior cancer did not affect the risk estimate	Age; calendar year; prior cancer; DM type

Currie et al, 2009 [6]	2000-?	General practice database (THIN)	General practice database (THIN)	Incident	≥ 1 insulin prescriptions	During follow-up: exposure status changes when a new drug of interest is prescribed.		6	Yes, any cancer	Age; sex; smoking status; diagnosis of a prior cancer *****
Fagot et al, 2013 [47]	2007-2010	Insurance database (SNIIRAM); reimbursements	Hospital discharge database (Programme de Medicalisation des Systemes d'Information (PMSI))	Incident	≥ 2 prescriptions of the same insulin type during 6 months period	At baseline, first prescription of an insulin type. Not censored if discontinued or switched	Median Glargine: 2.67 Determir: 2.75 HI: 2.83	12	Yes, any cancer	NIAD class; DM duration *****
Ferrara et al, 2011 [48]	1997-2005	Pharmacy database (dispensed)	Cancer registry of KPNC	Incident	≥ 2 prescriptions of the same ADD during 6 months period (ever user)	During follow-up: ever use (yes/no) changes over time	NR	6	Yes, any cancer	Age; year cohort entry; hba1c; DM duration; new DM diagnosis; smoking; ethnicity; income; creatinine; congestive heart failure; other DM medication
Gu et al, 2013 [50]	2001-2011	Diabetes register (SDR)	Shanghai Municipal Center for Disease Control and Prevention	Incident	≥ 6 months treatment with ADD	During follow-up: insulin use (yes/no)	Any human insulin: 3.37 No insulin: 4.23	0	Yes, any cancer	Age; hba1c; DM duration; smoking status; macrovascular disease; concomitant NIAD
Habel et al, 2013 [51]	2001-2009	Computerized outpatient pharmacy records; dispensed	Cancer registry of KPNC and KPSC	Incident	≥ 2 prescription of the same insulin type during 6 months period	During follow-up: ever use (yes/no) changes over time	Median Glargine: 1.2 NPH: 1.4 (full cohort)	0	Yes, any cancer	Age; site; year of entry; metformin; insulin *****
Hsieh et al, 2012 [53]	2002-2008	Insurance database (NHI); claims	Insurance database (NHI)	Prevalent	≥ 1 insulin prescription	During follow-up: exclusive users during whole follow-up period	NR	0	Yes, any cancer	Age

Kostev et al, 2012 [55]	2000-2011	Research database (IMS disease analyzer); prescribed	NR	Prevalent	≥ 1 insulin prescription	NR	NR	0	NR	Age; sex; hba1c; cumulative duration of exposure; private insurance status; urban location of practise; region; Charlson Comobidity Index
Lind et al, 2012 [56]	1985-2007	Clinical diabetes database (Diab-base)	Cancer registry and cause of death register	NR	≥ 1 insulin prescription	During follow-up (intervals): exposure status and duration were updated	Glargine: 3.5<br>Non-glargine: NR	0	Yes, breast cancer	Age; BMI; DM type; time since start follow-up; time since start glargine; last insulin dose used; smoking
Ljung et al, 2011 [57]	2006-2008	Pharmacy database (Swedish Prescribed Drug Register); dispensed	Cancer register and Cause of death register	Prevalent	≥ 1 insulin prescription during 6 months period	During fixed period (6 months), follow-up starts after this period	NR	6	Yes, breast cancer	Age; BMI; age at onset of diabetes; smoking; age at first child birth; oestrogen; cardiovascular disease
Morden et al, 2011 [59]	2006-2008	Insurance database; claims	Insurance database	Prevalent	≥ 1 insulin prescription during 4 months period	During fixed period (4 months) mutually exclusive groups were defined. Follow-up starts after this period	Glargine only: 1.9<br>Non-glargine insulin: 1.9	4	Yes, breast cancer	Age; obesity; smoking; insulin dose; metformin use; ethnicity; DM complications; estrogen; poverty; 14 Charlson comorbidities
Neumann et al, 2012 [60]	2006-2009	Insurance database (SNIIRAM); reimbursements	Hospital discharge database (Programme de Medicalisation des Systemes d'Information (PMSI))	Prevalent	≥ 2 prescription of insulin during 6 months period	During follow-up: insulin use (yes/no)	NR	6	Yes, breast and bladder cancer	Age; NIAD

Onitilo et al, 2014 [61]	1995-2011	Medical records (EMR)	Medical records (EMR) and cancer registry	Incident	≥ 1 ADD prescription	Time dependent follow-up: drug use (yes/no) changes over time	NR	0	Yes, breast and colon cancer	Age; BMI; date of DM diagnosis; hba1c; comorbidities; smoking history; insurance status; location of residence
Redaniel et al, 2012 [62]	1987-2007	General Practice Research Database (GPRD); prescribed	General Practice Research Database (GPRD)	Incident	≥ 6 months of continuous prescriptions for the same ADD class (insulin; sulfonylurea; metformin; other NIAD)	Time dependent follow-up: type and duration of drug use is determined over time, including drug overlap and prescription gaps. Treatment patterns were identified (mutually exclusive groups) by chronological order of drug prescriptions	NR	36; 0 in strata of duration	Yes, breast cancer	Age; BMI; period; region; year of diagnosed. In analysis stratified by duration of exposure: + weighted hba1c
Ruiter et al, 2012 [64]	2000-2008	Pharmacy database; dispensed	Hospital discharge records database (Dutch National Medical Register)	Incident	≥ 1 ADD prescription	During follow-up: duration of exposure. Exposure categories were mutually exclusive	Mean duration follow-up since 1st prescription Glargine: 2.2 Other insulin: 3.2 HI: 3.8	12	Yes, any cancer	Age; sex; calender time; other insulin types than exposure and comparison; nr of unique drugs used and nr of hospitalisations in the year prior to insulin start; prior NIAD use in days
Sturmer et al, 2013 [65]	2003-2010	Dispensed prescription medication claims captured in the MORE registry	Health plan register (MORE)	Incident	≥ 2 prescription of the same insulin type during 6 months period	At baseline, censored if discontinued or switched	Median Glargine: 0.9 NPH: 0.8	12	Yes, any cancer	Age; year of cohort entry; medications; comorbidities; hospitalizations; days in hospital; physician encounters; ED visits; screening tests
Suissa et al, 2011 [66]	2002-2009	General Practice Research Database	General Practice Research Database	Incident	≥ 1 insulin prescription	At baseline, not censored if discontinued or switched	NR	12	Yes, breast cancer	Age; obesity; hba1c; DM duration; excessive alcohol use; smoking status; oophorectomy; history of

		(GPRD); prescribed	(GPRD)						cancer; use of HRT; statins; other DM medication
Vallarino et al, 2013 [67]	2003-2010	Drug prescription claims captured in the i3 database	Health plan register (i3 database)	Incident	≥ 2 prescription for either pioglitazone or insulin during 6 months period	During follow-up: exclusive users (yes/no) during whole follow-up period	NR	6	Yes, any cancer	Inverse probability of treatment weights, i.e. propensity score (age, calendar year index date, obesity, medical conditions, NIAD, other medications)

*Not reported, therefore calculated by person years/n, ** used to evaluate potential information bias, *** used to evaluate potential confounding bias, **** minimum number of prescriptions during a specified period, ***** other covariates were assessed but not included in the final model as they had no impact on the risk estimate. Abbreviations: NR= not reported, DM= diabetes mellitus, HI= human insulin, ADD= anti-diabetic drugs, NIAD= non-insulin anti-diabetic drugs, HR= hazard ratio

ESM Table 3c. Characteristics of the randomized clinical trials included in the systematic review

Author, Year	Study period	Data source exposure **	Data source outcome**	Prevalent /incident user**	Exposure definition****	Mean duration of exposure (years)**	Latency period**	Exclusion of patients with a history of cancer	Covariates***
Bordeleau et al, 2014 [42]	2003-2011	RCT database	Cancer requiring hospitalization was collected and patients were asked retrospectively about cancer not requiring hospitalisation.	Prevalent	Glargine arm: glargine once daily, standard care arm: treated on the basis of the investigators best judgement	Trial of 6 years Glargine and standard care: mean: 5.6*, median: 6.2. Mean glargine adherence was 87.1%, in the standard care group 11% used non-glargine insulin	36	Patients with an expected survival <3 years are excluded	Treatment allocation at randomization; DM status at baseline; previous CV disease status; smoking; use of metformin and sulfonylurea. Age, DM duration, BMI, prior NIAD use and fasting plasma glucose were similar between treatment arms.
Dejgaard et al, 2009 [46]	NA, different per RCT	IPD (Novo Nordisk)	Adverse event databases from each RCT	NR	Determir arm; glargine or NPH as comparator arm	Determir vs. NPH trial median: 0.46 years; Determir vs. glargine median: 0.98 year	NR	NR	Age, DM status, DM duration, BMI and HbA1c were similar between the treatment arms
Home and Lagarenne 2009 [52]	NA, different per RCT	Pharmacovigilance database (sanofi-aventis)	Pharmacovigilance database (sanofi-aventis)	NR	glargine arm and 'any anti-diabetic drug' arm	Most studies: 0.5 years Glargine 0.8* Any anti-diabetic drug 0.9*	NR	NR	NR, different per RCT
Rosenstock et al, 2009 [63]	2001-2007	RCT database	Adverse events were reported by the investigator, as routine safety monitoring	Prevalent	glargine arm: glargine once daily, NPH arm: NPH twice daily	Trial of 5 years Glargine and NPH: 4.2* Prior exposure any insulin (% exposed; duration in years) glargine group: 67%, 5.5 NPH group: 70%, 4.9	0	No	NR; Age, DM status, DM duration, BMI, NIAD duration, prior insulin use, HbA1c and fasting plasma glucose were similar between the treatment arms

* Not reported, therefore calculated by person years/n, ** used to evaluate potential information bias, *** used to evaluate potential confounding bias, **** minimum number of prescriptions during a specified period. Abbreviations: NR= not reported, NA= not applicable, DM= diabetes mellitus, NIAD= non-insulin anti-diabetic drugs

ESM Table 4. Relative risk estimations for breast cancer among different duration and dose categories within insulin treatment groups

Author year	Exposure	Comparator	Breast cancer (n) exposure	Breast cancer (n) comparator	Definition of duration	Definition of dose	Category	Risk estimate*	95 % CI
Duration									
Insulin - NIAD: Hazard Ratio									
Redaniel et al, 2012c [62]	Insulin only users	Sulfonylurea only users	NR per category, 8 total	NR per category, 93 total	Duration since start exposure		<1 year	1.01	0.11-8.97
							1-5 years	0.54	0.18-1.68
							>5 years	2.25	0.72-6.99
Insulin - no insulin: Odds Ratio									
Bodmer et al, 2010 [41]	Insulin users	No insulin users	18	262	# of prescriptions, >40 reflects an exposure over 5 years		1-9	1.74	0.95-3.21
			11	262			10-29	1.30	0.62-2.70
			14	262			>29	1.51	0.76-3.01
Glargine – no glargine: Hazard Ratio									
Habel et al, 2013c [51]	Glargine only users	NPH insulin users	22	217	Duration since start exposure. Duration was calculated by adding the days between prescriptions		<2 years	1.2	0.7-1.9
			11	217			≥2 years	1.7	0.9-3.2
Lind et al, 2012b [56]	Glargine users	Non-glargine users	19	96	Hazard function of time since start of glargine		Per year	1.18	0.84-1.67
Sturmer et al, 2013b [65]	Glargine users	NPH users	37	7	Duration of drug use started from the second prescription until a patient stopped using the drugs or filled a prescription for another long-acting insulin		<6 months	0.99	0.46-2.13
			29	3			6-11 months	1.50	0.52-4.31
			26	6			12-23 months	1.09	0.38-3.12
			11	3			≥24 months	0.67	0.18-2.54
Suissa et al, 2011b [66]	Glargine users	Non-glargine insulin users	6	16	Duration since start exposure		<1 year	1.0	0.3-3.1
			8	23			1-3 years	0.9	0.3-2.7

			4<br>0	14<br>7			3-5 years<br>>5 years	0.8<br>NE	0.2-3.1<br>NE
Glargine – no glargine: Odds Ratio									
Grimaldi-Bensouda et al, 2013i [49]	Glargine users	Non-glargine users	NR	NR	Total duration of each insulin. The period of use was computed based on start/stop dates and switching		<4 years<br>4-7 years	1.15<br>0.94	0.70-1.88<br>0.51-1.74
Dose									
Glargine – no glargine: Hazard Ratio									
Fagot et al, 2013g [47]	Glargine users	Other int-/long-acting insulin only users	NR per category, 114 total	NR		Cumulative dose based on first insulin prescribed. Calculated by evenly distributing total dose of each insulin prescription over the days between the prescription date and the subsequent prescription date	<14000 IU<br>14000-27000 IU<br>>27000 IU	0.88<br>1.02<br>1.49	0.54-1.45<br>0.62-1.67<br>0.91-2.45
Lind et al, 2012c [56]	Glargine users	Non-glargine users	19	96		Hazard function of dose of glargine per Unit	Per unit	1.01	1.00-1.02
Morden et al, 2011c [59]	Glargine plus non-glargine insulin users	Non-glargine insulin users	NR	NR		Patients with mean daily dose in highest quartile	Highest quartile: 119 IU/day	1.00	0.57-1.76
Morden et al, 2011d [59]	Glargine only users	Non-glargine insulin users	NR	NR		Patients with mean daily dose in highest quartile	Highest quartile: 119 IU/day	1.75	1.10-2.78
Ruiter et al, 2012c [64]	Glargine only users	Human insulin only users	2<br>15	NR<br>NR		Stratified for median dose of first insulin	<median dose<br>=median	NE<br>1.22	NE<br>0.91-1.64

ESM Table 5. Quality evaluation of the epidemiological studies included in the systematic review*

**Author, Year**	**Bias**<br>Selection bias (1)	Information bias (2)	Confounding bias (3)	**Risk of bias** (4)	**Power** (5)
Bodmer et al, 2010 [41]	Time window bias; not matched on potential exposure time	Misclassification bias: no latency period	Not adjusted for physical activity, important risk factors for BC	High	Too low
Cleveland et al, 2012 [45]	Controls are only frequency matched to cases. Different participation rate among cases (82%) and controls (63%)	Recall bias: interview, no data on duration of exposure, misclassification bias: no latency period	Not adjusted for DM duration	High	Too low
Grimaldi-Bensouda et al, 2013 [49]	Survival bias: BC cases who survived 1-2 years		Not adjusted for physical activity	Low	Borderline
Koro et al, 2007 [54]	Controls were not sampled time-dependently: controls did not have a BC record at any time during their follow-up	Misclassification bias: no latency period, no data on duration of exposure	Not adjusted for BMI, DM duration, other DM medication, physical activity, important risk factors for BC	High	Too low
Mannucci et al, 2010 [58]		Misclassification bias: insulin was not necessarily initiated at start of follow-up, misallocation of exposure time: follow-up for cases included unexposed time as time is counted from start follow-up, while for controls exposure time is counted from actual start of insulin exposure.	Not adjusted for DM duration, physical activity, important risk factors for BC	High	Too low
Carstensen et al, 2012 [43]			Not adjusted for BMI, DM duration, other DM medication, physical activity, important risk factors for BC	Moderate	Adequate
Chang et al, 2011 [44]		Bias due to informative censoring: due to exclusive users design, switchers are excluded	Not adjusted for BMI, DM duration, physical activity, important risk factors for BC	Moderate	Too low
Colhoun et al, 2009 [5]		Intention-to-treat approach, no data on duration of exposure	Not adjusted for BMI, DM duration, other DM medication, physical activity, important risk factors for BC	High	Too low
Currie et al, 2009 [6]		No data on duration of exposure	Not adjusted for other DM medications, physical activity, important risk factors for BC	Moderate	Too low

Fagot et al, 2013 [47]		Misclassification bias: not censored if discontinued or switched	Not adjusted for BMI, other DM medications, physical activity, important risk factors for BC	High	Too low
Ferrara et al, 2011 [48]		No data on duration of exposure before and during cohort	Not adjusted for BMI and physical activity, important risk factors for BC	Moderate	Adequate
Gu et al, 2013 [50]			Not adjusted for BMI, physical activity, important risk factors for BC	Moderate	Too low
Habel et al, 2013 [51]		Misclassification bias: no latency period	Not adjusted for BMI, DM duration, physical activity, important risk factors for BC	Moderate	Too low
Hsieh et al, 2012 [53]		No data on duration of exposure before and during cohort, bias due to informative censoring: due to exclusive users design, switchers are excluded	Not adjusted for BMI, DM duration, other DM medication, physical activity, important risk factors for BC	High	Too low
Kostev, 2012 [55] (Letter)		No information to identify potential risk of bias, misclassification bias: no latency period	Not adjusted for BMI, DM duration, other DM medication, physical activity, important risk factors for BC	High	Too low
Lind et al, 2012 [56]		Misclassification bias: no latency period	Not adjusted for DM duration, other DM medication, physical activity, important risk factors for BC	Moderate	Too low
Ljung et al, 2011 [57]		Intention-to-treat approach, no data on duration of exposure before and during cohort	Not adjusted for other DM medication, physical activity, important risk factors for BC	High	Low
Morden et al, 2011 [59]		Intention-to-treat approach, no data on duration of exposure before cohort	Not adjusted for DM duration, other DM medication, physical activity, important risk factors for BC	High	Low
Neumann et al, 2012 [60]		Immortal time bias, main study outcome: bladder cancer, no data on duration of exposure before and during cohort	Not adjusted for BMI, DM duration, physical activity, important risk factors for BC	High	Adequate
Onitilo et al, 2014 [61]		No data on duration of exposure, misclassification bias: no latency period, no proper exposure-comparison	Not adjusted for other DM medication, physical activity, important risk factors for BC	High	Too low
Redaniel et al, 2012 [62]			Not adjusted for other DM medication, physical activity, important risk factors	Low	Too low

Redaniel et al, 2012 [62]			Not adjusted for other DM medication, physical activity, important risk factors for BC	Low	Too low
Ruiter et al, 2012 [64]			No adjustment for BMI, DM duration, physical activity, important risk factors for BC	Moderate	Too low
Sturmer et al, 2013 [65]			Not adjusted for BMI, DM duration, physical activity, important risk factors for BC	Moderate	Too low
Suissa et al, 2011 [66]		Intention-to-treat approach	Not adjusted for physical activity, important risk factors for BC	Moderate	Too low
Vallarino et al, 2013 [67]		Bias due to informative censoring: due to exclusive users design, switchers are excluded	Not adjusted for age, BMI, DM duration, other DM medication, physical activity, important risk factors for BC	High	Low
Bordeleau et al, 2014 [42]		Not designed to study cancer outcome	No data on physical activity and important risk factors for BC. Other important covariates were similar at baseline among the treatment arms.	Low	Too low
Dejgaard et al, 2009 [46]		Misclassification bias: no latency period	No data on physical activity and important risk factors for BC. Other important covariates were similar at baseline among the treatment arms.	Low	Too low
Home and Lagarenne 2009 [52]		Misclassification bias: no latency period	NR	Moderate	Too low
Rosenstock et al, 2009 [63]		Not designed to study cancer outcome; misclassification bias: no latency period	NR; important covariates were similar at baseline among the treatment arms	Low	Too low

Abbreviations: NR= not reported, NE= not estimated, BC=breast cancer, DM= diabetes mellitus

(1) Evaluation of loss to follow-up in cohort studies and selection of appropriate exposure and comparison groups in cohort studies and cases and controls in case-control studies. If cases were not matched to controls on calendar time and potential exposure time, we considered if time window bias could be present.

(2) Evaluation of misclassification of exposure and outcome. It was determined whether exposure was measured cumulative over time, if investigators censored for switching or discontinuation of insulin treatment and whether a latency time was included.

(3) Evaluation of adequate dealing with important risk factors in the analyses.

(4) Risk of bias is summarized in low, moderate and high based on a (subjective) qualitative evaluation of selection, information and confounding bias.

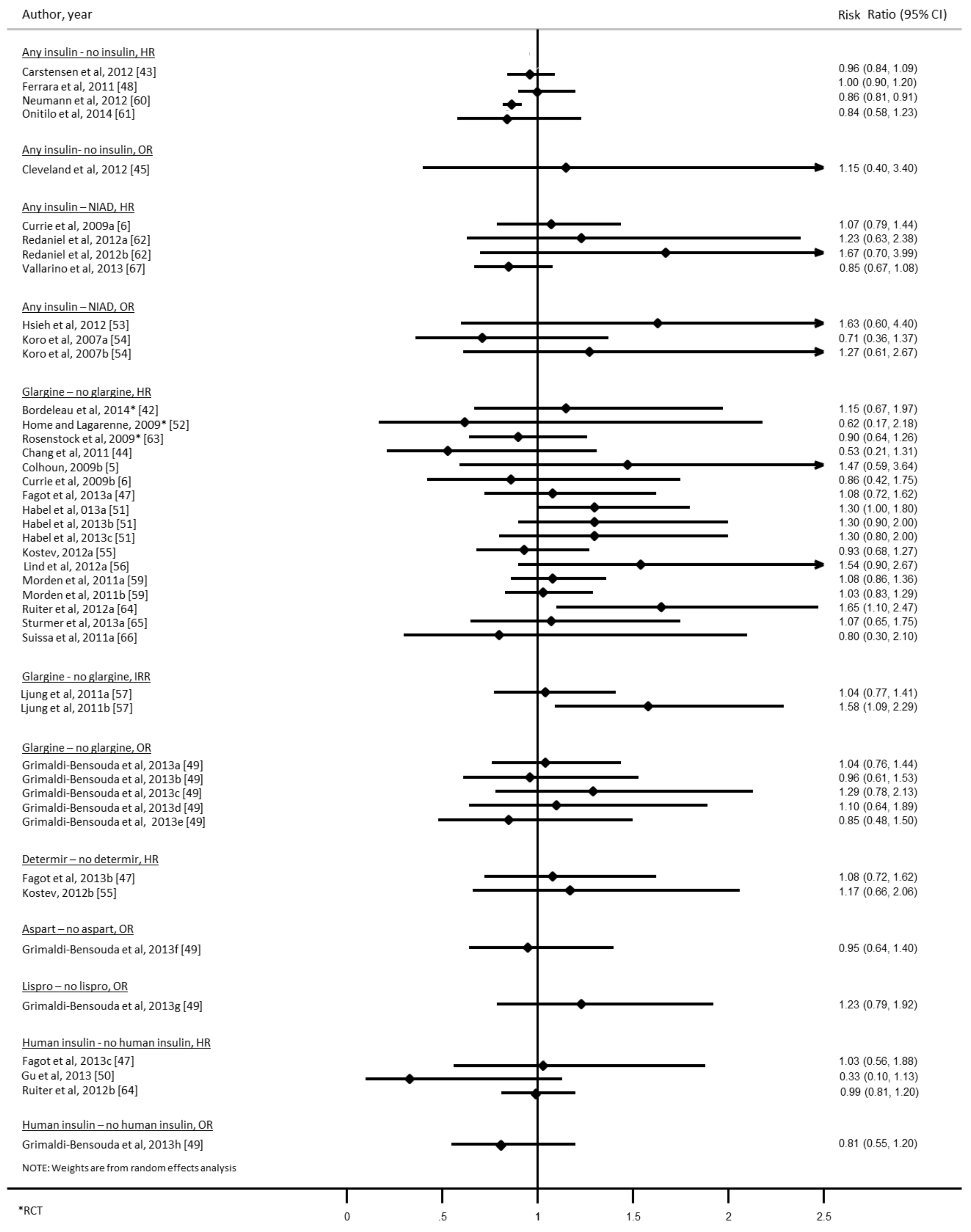

ESM Fig. 1. Forest plot of breast cancer risk among insulin (analogues) users stratified by treatment group and type of effect estimate
